# Long‐Term Temporal Divergence in Post‐Drought Resilience Decline Between Deciduous and Evergreen Tree Species

**DOI:** 10.1111/gcb.70330

**Published:** 2025-07-08

**Authors:** William Marchand, Claire Depardieu, Elizabeth M. Campbell, Jean Bousquet, Martin P. Girardin

**Affiliations:** ^1^ Institut National de l'Information Géographique et Forestière (IGN), Château des Barres Nogent‐sur‐Vernisson France; ^2^ Laurentian Forestry Centre Natural Resources Canada, Canadian Forest Service Québec Québec Canada; ^3^ Institut de Biologie Intégrative et des Systèmes Université Laval Québec Québec Canada; ^4^ Département des Sciences du Bois et de la Forêt, Centre d'Étude de la Forêt Université Laval Québec Québec Canada; ^5^ Pacific Forestry Centre Natural Resources Canada, Canadian Forest Service Victoria British Columbia Canada; ^6^ Centre d'Étude de la Forêt Université du Québec à Montréal Montréal Québec Canada; ^7^ Forest Research Institute Université du Québec en Abitibi‐Témiscamingue Rouyn‐Noranda Québec Canada

**Keywords:** dendroecology, drought stress, forest productivity, leaf habit, resilience indices, tree growth, croissance des arbres, dendroécologie, indices de résilience, productivité forestière, stress hydrique, type de feuillage

## Abstract

Severe drought increasingly threatens the resilience, productivity, and distribution of forest biomes worldwide. Understanding the evolution of tree drought resilience over the past century, along with its geographical and taxonomic relationships, is essential for predicting future forest dynamics. Using a tree‐ring database from Canadian forests, encompassing 40,147 trees across 4558 plots and 23 species, we analyzed temporal and spatial patterns of drought resilience. We examined how leaf habit, prior drought exposure, and site‐ and tree‐level factors influence growth resistance (immediate drought response), growth recovery (post‐drought growth resumption), and overall resilience. Our findings indicate that most major Canadian tree species exhibit low and declining drought resilience. Mean temperature, moisture availability, and elevation emerged as critical factors in shaping tree responses to drought. At high elevation, drought impacts were buffered by cool temperatures, enabling trees to maintain stable growth rates. Deciduous species showed a significant decline in recovery and resilience throughout the 20th century, whereas evergreen species displayed stable but low resilience and recovery. Summer droughts particularly reduced resistance and recovery in deciduous species compared to evergreens. However, prior drought exposure mitigated negative drought responses over a tree's lifetime, suggesting an adaptive capacity in both evergreen and deciduous species. Older forests unaccustomed to severe droughts appear especially vulnerable, potentially leading to shifts in ecosystem composition and reduced biodiversity. The declining resilience of deciduous species, combined with the low resilience of evergreens, suggests major changes for Canadian forests, including reduced productivity and altered species composition. Our results emphasize the importance of proactive forest management strategies to preserve forest productivity and biodiversity in the context of a changing climate.

## Graphical abstract

Severe drought increasingly threatens the resilience of forest biomes worldwide. Using a tree‐ring database from Canadian forests, encompassing 40,147 trees across 4558 plots and 23 species, we analyzed temporal and spatial patterns of drought resilience. Deciduous species showed a significant decline in resilience throughout the 20th century, whereas evergreens displayed stable but low resilience. At high elevation, drought impacts were buffered by cool temperatures. Summer droughts particularly reduced resistance and recovery in deciduous species compared to evergreens. Prior drought exposure mitigated negative drought responses over a tree's lifetime, suggesting an adaptive capacity in both evergreen and deciduous species.

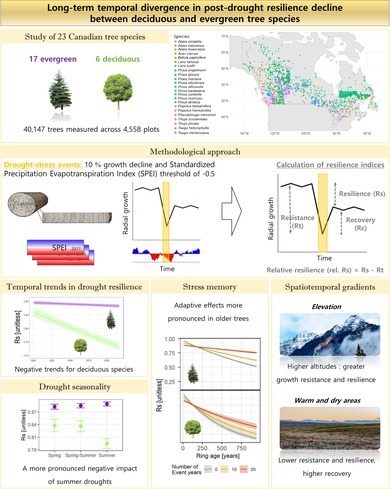

## RÉSUMÉ

Les sécheresses sévères menacent de plus en plus la résilience, la productivité et la distribution des biomes forestiers à l'échelle mondiale. Comprendre l'évolution de la résilience des arbres face à la sécheresse au cours du siècle dernier, ainsi que ses relations géographiques et taxonomiques, est essentiel pour prédire la dynamique future des forêts. En utilisant une base de données de cernes d'arbres provenant des forêts canadiennes, incluant 40,147 arbres répartis sur 4558 parcelles et 23 espèces, nous avons analysé les tendances spatio‐temporelles de la résilience à la sécheresse. Nous avons examiné comment le type de feuillage, l'exposition antérieure à la sécheresse et divers facteurs liés au site et à l'arbre influencent la résistance à la croissance (réponse immédiate à la sécheresse), la récupération de la croissance (reprise post‐sécheresse) et la résilience globale. Nos résultats indiquent que la plupart des principales espèces arboricoles canadiennes présentent une résilience à la sécheresse faible et en déclin. La température moyenne, la disponibilité en eau et l'altitude se sont révélées être des facteurs déterminants dans la réponse des arbres à la sécheresse. Les régions en haute altitude atténuent les impacts des sécheresses grâce à des températures plus fraîches, permettant aux arbres de maintenir des taux de croissance stables. Les espèces feuillues ont montré un déclin marqué de leur récupération et de leur résilience au cours du XXe siècle, tandis que les espèces résineuses ont affiché une résilience et une récupération stables, mais faibles. Les sécheresses estivales ont particulièrement réduit la résistance et la récupération des espèces feuillues par rapport aux conifères. Cependant, une exposition préalable aux sécheresses a atténué leurs effets négatifs au cours de la vie des arbres, suggérant une certaine capacité d'adaptation chez les espèces feuillues et résineuses. Les forêts âgées, peu habituées aux sécheresses sévères, apparaissent particulièrement vulnérables, ce qui pourrait entraîner des modifications dans la composition des écosystèmes et une réduction de la biodiversité. Le déclin de la résilience des espèces feuillues, combiné à la faible résilience des conifères, laisse entrevoir des changements majeurs dans les forêts canadiennes, notamment une diminution de la diversité des espèces et une redistribution des biomes forestiers. Nos résultats soulignent l'importance de stratégies de gestion forestière proactives pour préserver la productivité et la biodiversité des forêts dans un contexte de changement climatique.

## 1 Introduction

Ongoing climate change is threatening the integrity of natural ecosystems worldwide (IPCC 2023), with forest ecosystems being particularly vulnerable. The adverse effects are already evident (Forzieri et al. 2022; Hammond et al. 2022), as a growing body of research reveals rising tree mortality rates (Chaudhary et al. 2023; Rebollo et al. 2024; Xu et al. 2024) and widespread canopy dieback among many economically and ecologically important tree species (Hartmann et al. 2022; Liu et al. 2023; van Mantgem et al. 2009). These declines have been closely linked to increasingly severe drought episodes—extended periods when soil moisture drops below the level required to sustain plant water needs, often coinciding with heatwaves (Andrus et al. 2024; Matusick et al. 2013). Drought stress can interact with other contributing factors to mortality, such as pest outbreaks and forest diseases (Büntgen et al. 2019), compounding its effects on tree health and survival (McDowell et al. 2018). Forests provide a wide range of ecosystem services essential to society, as they play central roles in the carbon and water cycles, the mitigation of anthropogenic greenhouse gas emissions, and the preservation of biodiversity (Gauthier et al. 2015; Reid 2005). With drought severity expected to intensify over the next century in many areas of Europe and South, Central, and North America (IPCC 2023; Vicente‐Serrano et al. 2022), enhancing our understanding of both past forest responses and potential future impacts of drought stress on forest health is crucial.

Considerable attention has been given to understanding and quantifying tree growth resilience to drought in the context of climate change (Lloret et al. 2011; Schwarz et al. 2020). Growth resilience is defined as the capacity of a drought‐stressed tree to reach growth rates similar to those displayed in the years before drought (Lloret et al. 2011). Tree species display a diverse range of growth resilience, influenced by variations in their growth responses and species‐specific functional traits that are essential for survival in different habitats (Aubin et al. 2016; DeSoto et al. 2020). Studies have highlighted the importance of leaf habits and lifespan in shaping the mechanisms underlying tree resilience to drought (Alonso‐Forn et al. 2021; Kaproth et al. 2023), which are closely linked to the trade‐offs among carbon costs, stress protection, and resource acquisition. While drought significantly impacts tree growth, the responses differ notably between angiosperms and gymnosperms (DeSoto et al. 2020; Gazol et al. 2016). Compared with angiosperms, gymnosperms, particularly conifers, key species in vast ecosystems, have evolved adaptations such as fewer stomata and thicker cuticles to reduce water loss, granting them superior drought resistance (Baldi and La Porta 2022). In high‐latitude boreal and cold‐temperate forests, angiosperms, or flowering trees, are frequently considered more susceptible to drought than gymnosperms, partly due to their more intricate hydraulic systems and the role of stem photosynthesis in preserving hydraulic function during dry periods (Natale et al. 2023; Trifilò et al. 2021). Leaf habit, which refers to whether a tree retains its foliage year‐round (evergreen) or sheds it seasonally (deciduous), is another important factor influencing tree drought sensitivity (Dai et al. 2025; Kaproth et al. 2023). However, studies conducted across various biomes have reported contradictory effects of deciduousness on drought resilience (Alonso‐Forn et al. 2021; Kaproth et al. 2023), underscoring the need for further research in Canada's vast forest ecosystems, where such patterns remain unexplored.

Tree drought sensitivity and resilience are shaped by factors operating at multiple scales, ranging from the individual tree level to the stand to the landscape (Serra‐Maluquer et al. 2022). Environmental gradients shape both the biogeographical range of species and their local distribution across landscapes. Within a given landscape, trees in arid environments typically exhibit lower resistance but higher resilience to drought than those in wet environments (Gazol et al. 2018; Sykes and Prentice 1996). Elevation adds complexity, potentially offering climate refugia at higher altitudes (Jiang et al. 2024; Stralberg et al. 2020; Marqués et al. 2016). Moreover, differences in geomorphology between high‐altitude sites and lowland areas can also influence tree drought resilience. For example, the closer proximity of trees in lowland areas to riparian zones may facilitate easier access to groundwater than for trees at higher elevation (Trumper et al. 2024). Stand‐specific soil properties and slope further influence the effects of drought (Barnett et al. 2005; Erickson et al. 2015; Zhao et al. 2022; Trouvé et al. 2017). Differences in forest structure and composition, whether due to contrasting management strategies or variations in tree species, can influence drought resilience by altering competition pressure for moisture and nutrients, and by modifying trees' exposure to dry conditions (Lucas‐Borja, Andivia, et al. 2021; Lucas‐Borja, Bose, et al. 2021; Liu et al. 2022). At the tree level, factors such as age and size can influence the effect of drought stress on tree growth, with old and large trees generally being less resilient and resistant to drought than young, small trees (Martínez‐Vilalta et al. 2012; Zang et al. 2014), possibly due to reduced photosynthetic capacity or changes in root structure of old trees (Rozas et al. 2009; Yoder et al. 1994). However, other studies report tall, old trees of some species are more resistant to drought (Carnwath and Nelson 2017), possibly related to extensive root systems enabling access to limited soil moisture. In contrast, tree height can also negatively influence drought tolerance (Bennett et al. 2015; McGregor et al. 2021), as taller, dominant trees are more vulnerable to embolism because they have wider conduits compared to smaller, understory trees (Olson et al. 2018; Pretzsch et al. 2018). These multiscale factors interact in complex ways, making it challenging to pinpoint universal drivers of tree resilience to drought, particularly in Canada, which, spanning almost 90° in longitude and 30° in latitude, displays highly diverse climatic conditions and forest ecosystems (Aubin et al. 2018).

Environmental gradients also shape drought resilience more indirectly by driving the evolutionary selection of functional traits (González de Andrés et al. 2024; Silvestro et al. 2023). Different species, populations, and even individual trees within the same population present distinct traits that optimize trade‐offs between carbon acquisition and water conservation, influencing their drought resilience under specific environmental conditions (Aranda et al. 2012; Brendel and Epron 2022; Depardieu et al. 2024). However, a major challenge lies in distinguishing between biotic, trait‐driven effects and the influence of environmental gradients on tree drought resilience, as these factors often co‐vary (Serra‐Maluquer et al. 2022).

The timing of droughts and prior drought exposure are as important as environmental factors in explaining variations in tree growth resilience to drought (Bose et al. 2021; M. Huang et al. 2018). Spring droughts are particularly harmful to temperate and boreal trees as they coincide with the resurgence of cambial activity, a process critical for water availability and leaf development in broadleaf trees (González and Eckstein 2003). For evergreen conifers, spring droughts can hinder photosynthesis and early‐season growth. On the other hand, summer droughts can affect both broadleaf and conifer species by reducing growth rates, shortening the growing season, and preventing the replenishment of carbohydrate reserves needed for survival and resilience (D'Andrea et al. 2021; Oberleitner et al. 2022; Weemstra et al. 2013). In boreal ecosystems, short growing seasons may amplify the adverse effects of summer droughts. Over time, repeated severe droughts compromise the hydraulic system and carbohydrate storage of both functional groups, though their physiological responses and recovery capacities may differ (Bréda et al. 2006). This heightened vulnerability increases the risk of mortality during subsequent droughts. Indeed, recent studies have linked declining drought resilience with increased mortality rates (Cabon et al. 2023; DeSoto et al. 2020). In addition to the immediate effects of droughts, post‐drought effects, known as drought legacies, can persist long after a drought has ended (Vilonen et al. 2022). Trees in boreal forests may be especially vulnerable to significant legacy effects, as the short growing season limits their ability to fully replenish carbohydrate reserves and repair damaged vessels (Itter et al. 2019). While the importance of drought legacies has gained attention in recent years (Anderegg et al. 2020b; Müller and Bahn 2022; Sterck et al. 2024), our understanding of how they influence tree resistance and recovery to subsequent drought events is incomplete (Gessler et al. 2020).

In this study, we analyzed a subcontinental dataset of annual radial growth increments from 1901 to 2017, covering 23 tree species (6 deciduous and 17 evergreen) across Canadian forests. To assess tree responses to severe drought events, which are defined as periods of significant water stress causing noticeable growth reductions (Boken et al. 2005), we quantified four drought resilience indices, namely resistance, recovery, resilience, and relative resilience. Our study explored how leaf habit, drought recurrence with varying seasonality and intensity, and environmental gradients shape tree drought resilience. We first compared resilience indices between deciduous and evergreen species, and then examined variations across geographic and climatic gradients. Based on previous research (Anderegg et al. 2015; DeSoto et al. 2020; Gazol et al. 2016), we hypothesized that evergreens experience greater drought‐induced growth reductions than deciduous species and that their resilience capacities diverge over time due to increasing drought frequency. Given contrasting spatial resilience patterns between gymnosperms and angiosperms (Serra‐Maluquer et al. 2022) and trade‐offs in resistance and resilience occurring in conifers (X. Li et al. 2020; Zheng et al. 2023), we expected evergreens to exhibit distinct spatial and temporal resilience trends. Finally, we hypothesized that successive droughts would have cumulative effects that weaken growth resilience and delay or limit recovery capacity.

## 2 Materials and Methods

### 2.1 Growth Data

**FIGURE 1:**
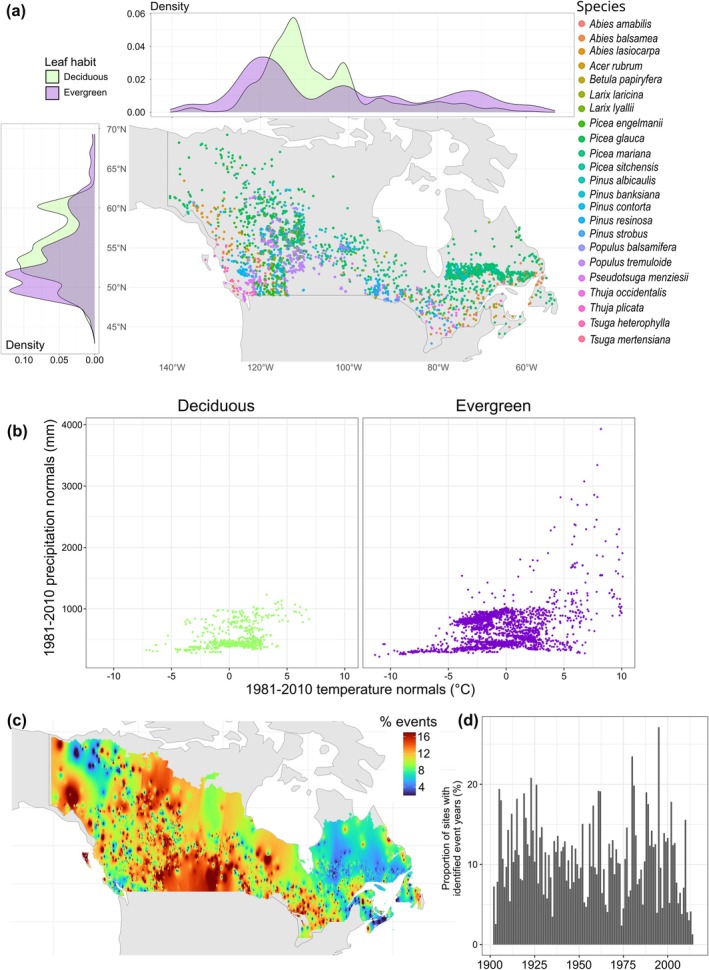
Distribution of the sampled data. Sample plots across Canada (a) and within the climate space (b). The percentage of sites (%) for which a drought‐stress event coupled with a growth decline was identified relative to the total number of sites, by location (c) and calendar year (d). The distribution of drought‐stress event dates by leaf habit is also illustrated with horizontal and vertical probability density plots in (a). In these plots, the “density” axis represents a probability density, meaning that the total area under each curve equals 1. Here, the unit of the density axis is the inverse of the *x*‐axis unit. For (c), an inverse‐distance weighting interpolation of 0.01° resolution was performed across 100 sampling sites. Map lines delineate study areas and do not necessarily depict accepted national boundaries.

We used data from the Canadian Forest Service tree‐ring data (CFS‐TRenD) repository. This database contains tree‐ring records from 40,147 trees across 4558 plots. These records were compiled from various sources including provincial and national inventories, as well as the International Tree‐Ring Data Bank (see Girardin et al. (2021) for details). Throughout this manuscript, we use the term “plot” to refer to all sampling locations, although we acknowledge that a subset of the data was collected at the stand level rather than within formally established plots. Our analysis focused on annual growth increments from 23 economically and ecologically significant tree species, including four broadleaved deciduous tree species (*Acer rubrum, Betula papyrifera, Populus balsamifera, Populus tremuloides*), two coniferous deciduous species (*Larix lyalii, Larix laricina*), and 17 coniferous evergreen species (Table 1, Figure 1, Figures S16 and S17). The study area spans 26° of latitude and 87° of longitude, covering most of Canada. The climate gradient ranged from arid‐cold environments with < 500 mm of precipitation and an average annual temperature below −10°C to wet‐warm environments with annual precipitation totals above 1000 mm and an annual average temperature above 8°C. The tree‐ring measurements spanned from the year 572 to 2017 (Figure S1), with notable variations in the distributions of breast height diameter (dbh) and age (Figures S2 and S3). Ring width measurements were converted into basal area increments (BAIs) using the *bai.out* function from the *dplR* package in RStudio (Bunn et al. 2023; R Core Team 2023). BAI reflects the actual increase in the tree's cross‐sectional area at the stem base, which is a direct measure of growth that accounts for tree size. This calculation, which assumes perfectly circular cross‐sections, makes BAI a reliable proxy for secondary growth (Biondi and Qeadan 2008). However, a recent study found that age‐ and size‐related trends can still persist in BAI series (Klesse and Bigler 2025). To address this issue, we systematically included variables accounting for tree age and size in our models (see Section 2.4).

**TABLE 1:** Details of the tree species studied, the number of trees studied per species, and the number of drought‐stress events identified and examined in this study are based on tree‐ring growth chronologies and climate data.

Species ID	Species Latin name	Common name	Leaf habit	Number of plots	Number of trees	Mean tree age (min–max)	Climatic drought occurrences (SPEI)	Growth drops (radial growth)	Number of drought‐stress events (climate and growth)	Average number of drought‐stress events per tree and 20‐year period
ABIEAMA	*Abies amabilis*	Pacific silver fir	Evergreen	20	212	191 (9–631)	771	327	213	1.9
ABIEBAL	*Abies balsamea*	Balsam fir	Evergreen	205	842	70 (4–248)	5230	2443	1522	1.9
ABIELAS	*Abies lasiocarpa*	Subalpine fir	Evergreen	93	932	161 (14–507)	3654	1661	1066	2.1
ACERRUB	*Acer rubrum*	Red maple	Deciduous	48	138	52 (8–136)	774	441	306	2.2
BETUPAP	*Betula papiryfera*	Paper birch	Deciduous	182	661	82 (7–258)	3666	2696	1781	2.5
LARILAR	*Larix laricina*	Tamarack	Deciduous	177	598	90 (4–334)	3881	3131	1858	2.6
LARILYA	*Larix lyallii*	Subalpine larch	Deciduous	24	562	350 (67–734)	1120	699	408	2.7
PICEENG	*Picea engelmannii*	Engelmann spruce	Evergreen	130	1667	201 (6–575)	5202	2261	1461	2.0
PICEGLA	*Picea glauca*	White spruce	Evergreen	673	4683	142 (4–631)	21,562	12,812	8327	2.3
PICEMAR	*Picea mariana*	Black spruce	Evergreen	1993	9945	118 (5–598)	70,785	32,719	19,303	2.1
PICESIT	*Picea sitchensis*	Sitka spruce	Evergreen	10	205	160 (20–306)	429	129	76	2.0
PINUALB	*Pinus albicaulis*	Whitebark pine	Evergreen	19	449	354 (22–983)	703	236	141	1.6
PINUBAN	*Pinus banksiana*	Jack pine	Evergreen	730	4391	75 (4–327)	15,724	10,083	6011	2.3
PINUCON	*Pinus contorta*	Lodgepole pine	Evergreen	282	2935	102 (6–489)	11,094	5232	3265	2.2
PINURES	*Pinus resinosa*	Red pine	Evergreen	47	869	128 (28–397)	2014	1020	679	2.5
PINUSTR	*Pinus strobus*	Eastern white pine	Evergreen	55	852	142 (10–407)	2194	1031	669	2.2
POPUBAL	*Populus balsamifera*	Balsam poplar	Deciduous	130	362	61 (4–215)	2413	2005	1377	2.6
POPUTRE	*Populus tremuloides*	Trembling aspen	Deciduous	778	3904	65 (3–243)	19,704	15,261	8983	2.4
PSEUMEN	*Pseudotsuga menziesii*	Douglas fir	Evergreen	232	2803	136 (13–802)	9002	4967	2958	2.9
THUJOCC	*Thuja occidentalis*	Northern white‐cedar	Evergreen	56	769	167 (19–745)	2315	942	620	1.7
THUJPLI	*Thuja plicata*	Western red cedar	Evergreen	35	177	136 (11–650)	946	665	369	2.1
TSUGHET	*Tsuga heterophylla*	Western hemlock	Evergreen	54	232	108 (10–603)	1863	1222	715	2.3
TSUGMER	*Tsuga mertensiana*	Mountain hemlock	Evergreen	16	143	219 (56–571)	664	389	227	2.2

*Note:* The correspondence between the species IDs used throughout the manuscript, Latin names, and common names are presented. For each species, the number of drought‐stress events detected from climate data (column “climatic drought occurrence”), from growth variations (column “growth drops”), and from combining climate data and radial growth (column “number of drought‐stress events”) are reported. To identify drought events, the 3‐month standardized precipitation‐evapotranspiration index (SPEI) for either May (spring drought) or August (summer drought) was used. “Growth drops” were defined as years in which the basal area increment (BAI) of at least 75% of the trees in a plot was at least 10% lower than in the preceding year.

### 2.2 Climate Data and Characterization of Drought‐Related Growth Declines

The climate data for each plot, spanning the period from 1901 to 2017, were obtained using the BioSIM v.11 software (Régnière et al. 2017). BioSIM interpolates daily climate data by using records from the four nearest weather stations and adjusts for differences in elevation. The daily mean temperature and total precipitation were aggregated to a monthly scale. The standardized precipitation evapotranspiration index (SPEI) was subsequently calculated, considering the cumulative precipitation‐evapotranspiration over a 3‐month period (3‐month SPEI) using the R‐package *spei* (Beguería and Vicente‐Serrano 2023). The 3‐month SPEI was selected because it effectively captures the seasonal moisture dynamics most relevant to tree growth while avoiding the dampening effect of indices calculated over several seasons that can obscure critical short‐term climatic variations. The choice of this parameter ensures a more accurate representation of water availability and its direct impact on tree growth in boreal forests, compared to longer aggregation periods. In addition, to capture regional climate gradients, temperature (MAT) and precipitation (MAP) normals for the period 1981–2010 were calculated.

Drought‐stress event years were identified on the basis of both the SPEI and growth rates by plot and tree species (Figure S14). A year was classified as a drought‐stress event year if the corresponding SPEI value (either May in the case of spring drought or August in the case of summer drought) was < −0.5 and if the population of trees at the plot location experienced a significant reduction in the growth rate (BAI) that year. The choice of −0.5 as a threshold corresponds to approximately the 30th percentile of the standard normal distribution, implying that conditions are drier than usual in roughly 30% of cases. We selected this threshold to detect the onset of dry conditions, as even mild drought can influence tree growth, especially in cold or moisture‐limited environments such as those found across much of Canada. Additionally, years where reduced growth was observed immediately following a drought year were also considered drought‐stress event years. Growth reductions were determined using the function *res.comp* of the R package *pointRes* v.1 (van der Maaten‐Theunissen et al. 2015). A year was flagged for further investigation if the BAI of at least 75% of the trees of a plot was 10% lower than the growth of the year immediately preceding it. The 10% threshold, previously used in a study by Sohn et al. (2016), represents a relatively low benchmark for BAI decline, enhancing the likelihood of detecting any stressors with a significant impact on tree growth at the population level. If one of these conditions was not met, the year was not considered a drought‐stress event. We excluded years with SPEI < −0.5 that did not result in reduced BAI to focus on years where drought conditions had a tangible negative effect on tree growth. This filtering ensures that resistance values capture genuine drought impacts, rather than coincidental growth increases driven by other climatic factors. In northern or hydric sites, low SPEI values can sometimes align with favorable conditions—such as longer growing seasons or decreased soil saturation—that promote growth despite a negative water balance. Including such years would bias resistance values upward, as trees may appear drought‐resistant while actually benefiting from these conditions. This methodological choice explains why our resistance values are systematically below 1.

Autocorrelation in tree‐ring width series (Cook and Kairiukstis 1990) introduces uncertainty in resilience estimates by smoothing growth fluctuations and dispersing drought impacts over multiple years (Klesse et al. 2023; Esper et al. 2015). To assess its effect on detecting drought‐induced growth reductions and, consequently, on the inclusion of a subset of trees exhibiting significant growth declines during dry spells in our analyses, we performed Cohen's d tests on first‐order autocorrelation (AR1) using the *acf* function (*stats* R‐package; see Methods S1 for details). Comparing trees with and without detected drought‐stress events (*rstatix* package; Kassambara 2023), we found negligible effect sizes (|*d*| < 0.1), indicating that autocorrelation did not bias tree selection (Figure S4, Table S1). Thus, our sample remains representative and unbiased in defining drought‐stress years based on negative SPEI and growth reductions.

### 2.3 Computing Resilience Indices

The resilience indices, including resistance (Rt), recovery (Rc), resilience (Rs), and relative resilience (rel. Rs), were calculated following Lloret et al. (2011) based on tree growth rates. Resistance (Rt) represents a tree's ability to maintain growth during drought, measured as the ratio of BAI during drought (Dr) to pre‐drought BAI (PreDr). Recovery (Rc) reflects a tree's capacity to restore growth post‐drought, calculated as PostDr/Dr, where PostDr is the BAI after drought. Resilience (Rs) measures a tree's return to pre‐drought growth, expressed as PostDr/PreDr. Relative resilience (rel. Rs) assesses recovery in relation to the initial drought impact, calculated as Rs minus Rt. A positive rel. Rs value indicates that recovery surpasses initial stress effects, highlighting strong recovery capacity. Since extreme drought‐stress events can affect tree growth for several years (Anderegg et al. 2015), we calculated the four indices considering pre‐drought and post‐drought periods ranging from 2 to 8 years. To avoid the confounding effects of overlapping drought‐stress events identified in our study, we excluded resilience indices where pre‐ or post‐drought periods overlapped with another drought‐stress event.

Bivariate plots and smoothing regression lines (either linear regressions or generalized additive models) were used to visually assess the interrelationships among the four resilience indices. To assess potential trade‐offs between resistance (Rt) and recovery (Rc), we fitted a negative exponential function, which provided the best fit to our data. We further explored the integrated assessment of the Lloret indices by comparing the negative exponential fit to a hypothetical line representing full resilience, where recovery fully compensates for drought‐induced growth reductions. This line of full resilience was generated using the original formula of Lloret et al. (2011), which was modified to set the resilience index to 1, as proposed by Schwarz et al. (2020):

(1) $${\mathrm{Rc}}_{\text{full}}=1/\mathrm{Rt},$$

where Rc_full_ is a hypothetical Rc value for which, at a given observed Rt value, the drought resilience would be strictly equal to 1. By comparing the regression fits for the relationship between Rt and Rc from our data with the theoretical line of full resilience, the two taxonomic groups (deciduous vs. evergreens) and the studied tree species can be assessed and ranked (Schwarz et al. 2020).

### 2.4 Statistical Analyses

To visualize the spatial distribution of drought stress events, we first used the Spatial Analyst tool in ArcMap to perform an inverse‐distance weighting interpolation (0.01° resolution) based on the proportion of detected events across 100 sampling sites. We then developed two sets of linear mixed‐effects models: the first set focused on evaluating resilience indices at the species level (“species models”; Figures S8–S11), while the second assessed these indices by grouping species according to their leaf habit (“leaf habit models”). In both sets, the response variables were either Rt, Rc, Rs, or rel. Rs, each log‐transformed to ensure that the residuals followed a normal distribution. Resilience indices values were calculated for each tree and drought‐stress event year individually, rather than being aggregated at the plot level, meaning that multiple values were generated per tree. Since exploratory analyses revealed that the results varied depending on the length of the time window (Tables S3–S6; see also Bose et al. (2024); Szejner et al. (2020); and Schwarz et al. (2020)), we incorporated values derived from all time windows into our analyses, treating the time window length as a random effect in the models. In our study, the timing of reduced growth could have occurred either during the drought year itself or in the subsequent year. To account for this variation, we included a 2‐level factor variable (timing of growth decline) as a random effect in our models (Table S2). The two sets of models differed in their explanatory variables. In the species models, we were interested in comparing the resilience capacities of all the tree species. We thus incorporated species identity as an explanatory variable, without additional plot‐level environmental factors, and did not perform model selection. Ring age and tree size were included to control for ontogeny‐related variability in tree drought resilience. The models included nested random effects accounting for time window length, timing of growth decline, and plot identity. The species model structure was as follows:

(2) $$\begin{array}{c} {\text{Index}}_{\mathrm{ijt}}\sim \text{Species}\;{\mathrm{ID}}_{i}+\text{Ring}\;{\mathrm{Age}}_{\mathrm{ijt}}+{\text{Size}}_{\mathrm{ijt}}\\ +\left(\text{Plot}\;\mathrm{ID},\text{TimingGrDecl},\text{Time window}\right)+\varepsilon_{\mathrm{ijt}}, \end{array}$$

where Index_*ijt*_ refers to the log‐transformed resilience index (Rt, Rc, Rs, or rel. Rs) of tree *i* in plot *j* at year *t*; Species ID_*i*_ is the species identity of tree *i*; Ring Age_*ijt*_ and Size_*ijt*_ are the cambial age of tree ring and tree size at time *t*, respectively, and (Plot ID, TimingGrDecl, Time window) refers to the random effect of the time window (the window chosen for the computation of resilience indices, ranging from 2 to 8 years) nested within the timing of growth decline (TimingGrDecl) nested within the plot identity (Plot ID). *ε* represents the residual error. To display the results, least‐square means, with 95% confidence intervals, were computed using the *emmeans* function of the r‐package *emmeans* (Lenth et al. 2024) and back‐transformed.

The leaf habit models were fitted to explore ecological and environmental drivers of tree capacity to cope with drought stresses. We selected explanatory variables based on a literature search (see Table S2) and a priori hypothesis testing. We specifically incorporated cambial age, basal area, leaf habit (categorized as evergreen or deciduous), latitude, longitude, elevation, temperature (MAT) and precipitation (MAP) normals, drought season (categorized as spring, summer, or both spring and summer drought), the number of prior drought‐stress events at each plot, and April–September SPEI to capture variations in drought intensity. Continuous explanatory variables were standardized by subtracting the mean and dividing by the standard deviation, producing standardized slope coefficients that facilitated comparisons of effect magnitudes across variables (Schielzeth 2010). Interaction terms were also chosen based on hypothesis testing and literature review (see Table 2). We performed model selection on interaction terms to keep the models as parsimonious as possible. Full models were those with two‐way interactions, as well as the three‐way interaction between leaf habit, ring age, and past drought exposure. We tested the significance of interaction terms by comparing AIC scores of a model including a given interaction term and a model excluding this term, starting with the less significant term based on the ANOVA table of the full model. Significant two‐way interactions of leaf habit with other explanatory variables, whose exclusion increased the model's AIC score by more than 2, were retained in the final models. The interaction between leaf habit, ring age, and the number of previous drought‐stress events, as well as the interaction between the number of previous drought stresses and the elevation, were also tested. Given that the timing of tree growth responses to drought can influence resilience, a factorial variable was included in the random effects, distinguishing between trees that responded in the year of drought occurrence and those with a lagged response. Variance inflation factors (VIFs) were examined to avoid multicollinearity, and interactions leading to a VIF > 10 were excluded. Continuous explanatory variables were only moderately correlated (see Figure S20), which means that even with relatively high VIFs, collinearity would not affect our conclusions substantially. The interaction terms were initially selected using the maximum likelihood (ML) method, while final models were fitted using restricted maximum likelihood (REML). Due to the significant data imbalance between the two leaf habit groups, a variance function was incorporated to separately estimate the variance for data from deciduous and evergreen trees. For each of the four resilience indices, the leaf habit models took the following form:

(3) $$\begin{array}{c} {\text{Index}}_{\mathrm{ijt}}\sim \text{Ring}\;{\mathrm{age}}_{\mathrm{ijt}}+{\text{Tree size}}_{\mathrm{ijt}}+{\text{SPEI}}_{\mathrm{jt}}+{\mathrm{MAT}}_{j}+{\mathrm{MAP}}_{j}+{\text{PrevDr}}_{\mathrm{ijt}}\\ +{\text{Elevation}}_{j}+{\text{DrSea}}_{\mathrm{jt}}+{\text{Year}}_{t}+{\text{Leaf habit}}_{i}+{\text{Leaf habit}}_{i}\times \text{Ring}\;{\mathrm{age}}_{\mathrm{ijt}}\\ +{\text{Leaf habit}}_{i}\times {\text{PrevDr}}_{\mathrm{ijt}}+{\text{Leaf habit}}_{i}\times {\text{DrSea}}_{\mathrm{jt}}+{\text{Leaf habit}}_{i}\times {\text{SPEI}}_{\mathrm{jt}}\\ +{\text{Leaf habit}}_{i}\times {\mathrm{MAT}}_{j}+{\text{Leaf habit}}_{i}\times {\mathrm{MAP}}_{j}+{\text{Leaf habit}}_{i}\times {\text{Elevation}}_{j}\\ +\text{Ring}\;{\mathrm{age}}_{\mathrm{ijt}}\times {\text{PrevDr}}_{\mathrm{ijt}}+{\text{Leaf habit}}_{i}\times {\text{Year}}_{t}+{\text{PrevDr}}_{\mathrm{ijt}}\times {\text{Elevation}}_{j}\\ +{\text{Leaf habit}}_{i}\times \text{Ring}\;{\mathrm{age}}_{\mathrm{ijt}}\times {\text{PrevDr}}_{\mathrm{ijt}}\\ +\left(\text{Plot}\;\mathrm{ID},\text{TimingGrowDecl},\text{time window},\text{species}\;\mathrm{ID}\right)\\ +\text{varIdent}\;\left(\sim 1\;|\;{\text{Leaf habit}}_{j}\right)+\varepsilon_{\mathrm{ijt}}, \end{array}$$

where Index_*ijt*_ represents the log‐transformed value of either Rt, Rs, Rc, or rel. Rs of tree *i* in plot *j* at year *t*; Ring age_*ijt*_ refers to the cambial age of tree *i* in plot *j* at year *t*, computed as the ring count; Tree size_*ijt*_ is the basal area of tree *i* in plot *j* at year *t*; SPEI_*jt*_ is the SPEI of plot *j* at year *t*; MAT_*j*_ and MAP_*j*_ are the temperature and precipitation normals for plot *j*; PrevDr_*ijt*_ represents the number of drought‐stress event dates previously experienced by tree *i* in plot *j* at year *t*; Elevation_*j*_ is the elevation at plot location *j*; DrSea_*jt*_ is the season of drought occurrence in plot *j* at year *t*, either spring, summer, or spring and summer, as a 3‐level factor variable; Year_*t*_ is the drought‐stress event year date; Leaf habit_*i*_ is the leaf habit of tree *i*, either deciduous or evergreen; a “×” symbol denotes an interaction term (See Table S2 for retained interaction terms); leafhabit_*i*_ × Ring age_*ijt*_ × PrevDR_*ijt*_ is the three‐way interaction term between leaf habit, ring age, and number of previous event dates; (Plot ID, Time window, TimingGrowDecl, species ID) refers to the random effect of species identity nested within the time window (the window chosen for the computation of resilience indices, from 2 to 8 years) nested within the timing of growth reduction (either the drought year or the following year) nested within the plot identity. varIdent(~1 | Leaf habit_*j*_) represents the variance function, and *ε* the residual error.

**TABLE 2:** Outputs from linear mixed models (Equation 2) for growth resistance (Rt), recovery (Rc), resilience (Rs), and relative resilience (rel. Rs) to drought.

Effect	Type	Resistance (Rt)		Recovery (Rc)		Resilience (Rs)		Relative resilience (rel. Rs)	
		*T*‐value	Signif.	*T*‐value	Signif.	*T*‐value	Signif.	*T*‐value	Signif.
(Intercept)	—	−60.2	***	36.1	***	−20	***	2267.5	***
Leaf habit	Fixed	29.4	***	−20.4	***	5.8	***	−13.3	***
Ring age	Fixed	−1	ns	−10.8	***	−15.4	***	−14.6	***
Tree size	Fixed	−0.2	ns	−5.6	***	−6.2	***	−4.9	***
Drought season in spring and summer (DrSea_spr_sum)	Fixed	−5.3	***	4.7	***	−0.2	ns	5	***
Drought season in summer (DrSea_sum)	Fixed	6.3	***	−10.2	***	−7.1	***	−9.6	***
Standardized precipitation evapotranspiration index (SPEI)	Fixed	5.5	***	−3.4	**	3.7	***	−2.9	**
Temperature normals (MAT)	Fixed	−14.7	***	10.8	***	−5.7	***	8.6	***
Precipitation normals (MAP)	Fixed	9.9	***	−6	***	4.7	***	−4.7	***
Number of previous drought events (Prev_Dr)	Fixed	5.9	***	−9.6	***	−5.7	***	−4.4	***
Elevation	Fixed	7.9	***	2.3	*	7.4	***	2.1	*
Year	Fixed	1.1	ns	−10.4	***	−11.4	***	−13.2	***
Leaf habit:Ring age	Fixed	−2	*	9.5	***	10.5	***	11	***
Ring age:Prev_Dr	Fixed	−2.2	*	13.8	***	14.4	***	14.5	***
Leaf habit:DrSea_spr_sum	Fixed	4.3	***	−3.5	***	0.5	ns	−4.3	***
Leaf habit:DrSea_sum	Fixed	−7.7	***	12	***	8	***	11	***
Leaf habit:SPEI	Fixed	−3.3	**	2.4	*	*NI*	*NI*	2.8	**
Leaf habit:MAT	Fixed	9.1	***	−8.1	***	*NI*	*NI*	−5.6	***
Leaf habit:MAP	Fixed	−4.7	***	3	**	−3	**	*NI*	*NI*
Leaf habit:Prev_Dr	Fixed	−7.4	***	6.2	***	−0.3	ns	−0.3	ns
Prev_Dr:Elevation	Fixed	−3	**	*NI*	*NI*	−2.4	*	*NI*	*NI*
Leaf habit:Year	Fixed	−2.1	*	10.4	***	10.1	***	13.1	***
Leaf habit:Ring age:Prev_Dr	Fixed	7.4	***	−12.1	***	−6.6	***	−9.4	***
Leaf habit:Elevation	Fixed	*NI*	*NI*	−2.5	*	−4.3	***	−2.1	*
Time window	Random	—	***	—	***	—	***	—	***
TimingGrowDecl	Random	—	***	—	***	—	***	—	***
Plot ID	Random	—	***	—	***	—	***	—	***
Species ID	Random	—	***	—	***	—	***	—	***
Conditional *R* ^2^			0.43		0.45		0.35		0.50
Marginal *R* ^2^			0.14		0.09		0.03		0.05

*Note:* *T*‐values and significance levels (ns: not significant, *p < 0.05, **p < 0.01, ***p < 0.001) are reported for fixed effects. For random effects, the significance levels obtained from likelihood ratio tests, which compare the full model with the model excluding the random term of interest, are provided. At the bottom of the table, the marginal *R*‐squared value, which considers only the variance of the fixed effects, and the conditional *R*‐squared value which takes into account both the fixed and random effects (i.e., the total model), are reported for the four resilience indices. The reference levels for the factors were as follows: leaf habit: deciduous, drought season: spring. “NI”: interaction term not included because its removal did not increase the AIC by more than two, following model selection based on AIC scores. “—”: information not reported because it is not applicable.

We assessed the impact of ring age, climate‐ and topography‐related variables, and leaf habit on growth resilience indices using the *t*‐statistics of the parameter estimates, along with their associated *p* values for significance. The null hypothesis “no significant effect of the tested variable on the growth resilience index” was rejected when the *p* value was lower than 0.050. Linear mixed models were fitted using the *nlme* package version 3.1‐163 (Pinheiro et al. 2019) in R 4.3.1 (R Core Team 2023). Visual inspection of the residuals confirmed that the assumptions of homoscedasticity and normality were met (Figure S5).

To explore the effect that long‐term tree growth performance has on tree resilience to drought, we used a similar linear mixed‐model approach that tested the relationship between tree‐level averages of each resilience index and tree cumulative growth, represented by diameter at breast height (DBH; see Methods S2). This analysis was motivated by the hypothesis of a growth‐longevity tradeoff, with slow‐growing trees investing more in defense mechanisms, including drought‐related traits.

## 3 Results

### 3.1 Spatial and Temporal Distributions of Canadian Drought‐Stress Events

Among the 390,950 sample years, 41,429 drought years were characterized by a significant decline in tree growth (Figure 1c,d). These drought‐stress events were most frequent in the northernmost region of the Coast Mountains in the west, the Yellowknife region in the Northwest Territories, and the southern climatic region of the Prairies (Figure 1c). Temporally, they were concentrated in the late 1910s, early 1920s, and from the 1980s to the 2000s (Figure 1d). Only 4.7% of the tree samples were excluded from our analyses due to an absence of growth response to drought, primarily in conifer species such as *Picea mariana* , *Picea glauca* , and *Pinus banksiana* . Across all the species, trees experienced an average of 2.3 drought‐stress events each per 20‐year time period, ranging from 1 to 10 events. The average number of event years per tree and 20‐year period varied by species, from 1.6 in *Pinus albicaulis* to 2.9 in *Pseudotsuga menziesii* (Table 1). Drought‐stress events were most frequent in *Larix lyalii* (11.7% of the studied site‐years combinations per 20‐year period), *Populus balsamifera* (11.3%), and *Pseudotsuga menziesii* (10.9%), while *Abies balsamea* (5.9%) and *Thuja occidentalis* (5.8%) exhibited the lowest frequencies of drought‐stress event years per 20‐year period. The spatial distribution of drought‐stress event dates closely aligned with mapped drought‐sensitive areas in Canadian forests (Figure S6; see also Girardin et al. (2024)), confirming that our analysis effectively captured the impact of drought‐stress events on tree growth across the country.

### 3.2 Drought Resilience Across Leaf Habits: Temporal Variation and Relationships Between the Resilience Indices

**FIGURE 2:**
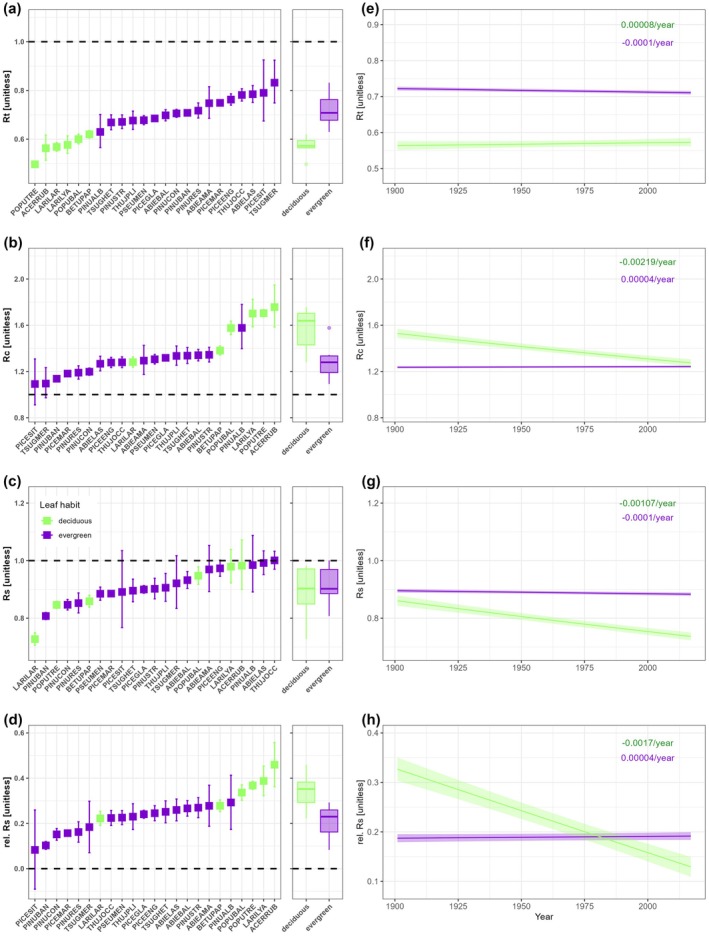
Estimated mean resilience indices for the study period and temporal variation based on leaf habit. (a–d) Least‐square model estimates of mean growth resilience indices by tree species, obtained from Equation (2). Means and error bars representing 95% confidence intervals are presented for growth resistance (Rt, a), growth recovery (Rc, b), growth resilience (Rs, c), and relative resilience (rel. Rs, d). Deciduous tree species are shown in green, while evergreen species are shown in violet. Boxplots in (a–d) display the distributions of model estimates for deciduous and evergreen species. (e–h) Predicted trajectories of growth resistance (e), recovery (f), resilience (g), and relative resilience (h) indices obtained from the models described in Equation (3), for evergreen and deciduous species. The predicted values were derived from linear mixed models, with shading indicating 95% confidence intervals. Statistically significant differences between time points can be inferred where confidence intervals do not overlap. Slope estimates for deciduous and evergreens are also provided. Dashed lines in (a–d) are intercepts of 1 (a–c) and 0 (d) that represent cases where the tree did not display any increase or decrease in growth in relation to drought stress events. Please refer to Table 1 for the Latin and common tree species names, Table 2 for details on the results of the leaf habit models (panels e–h), and Table S7 for detailed results of the species models (panels a–d).

The 23 species exhibited a mean resistance (Rt) significantly below one, indicating that their growth was generally reduced during drought‐stress events. In contrast, their recovery (Rc) was relatively robust, with mean values ranging from 1.01 to 1.95 (Figure 2). Both deciduous and evergreen species exhibited a broader range of Rc values compared to Rt values. Deciduous species generally showed lower resistance to drought than evergreens do. Among the deciduous species, *Populus tremuloides, Larix lyallii*, *Larix laricina* , and *Acer rubrum* had the lowest mean values (0.50–0.58), while *Populus balsamifera* and *Betula papiryfera* also exhibited notably low resistance, with Rt values of 0.6 and 0.62, respectively (Figure 2a). In contrast, evergreens demonstrated lower recovery capacities than deciduous species did; however, over time, the recovery rates of deciduous species tended to converge with those of evergreens (Figure 2f). Deciduous species generally exhibited higher Rc values than evergreens did, although the difference was less pronounced than that for Rt (Figure 2b). Interestingly, *Pinus albicaulis* ranked fourth among the six species with the highest Rc values, with the other five being deciduous. Among all the species, *Acer rubrum* showed the strongest recovery, with mean Rc value above 1.75, while *Picea sitchensis* and *Tsuga mertensiana* showed both high variability and the lowest mean Rc values. In terms of resilience (Rs), no significant differences were observed between deciduous and evergreen species (Figure 2c). *Populus tremuloides*, despite being the least resistant species, had the second‐highest mean recovery rate (Figure 2a,b). The least resilient species was *Larix laricina* , while the most resilient was *Thuja occidentalis* (Figure 2c). Deciduous species exhibited significantly greater relative resilience compared to evergreens, with *Acer rubrum* showing the highest mean rel. Rs value and *Picea sitchensis* the lowest (Figure 2d). Importantly, these variations in resilience indices were not linked to species‐specific differences in the temporal autocorrelation of tree‐ring data (Figure S7).

**FIGURE 3:**
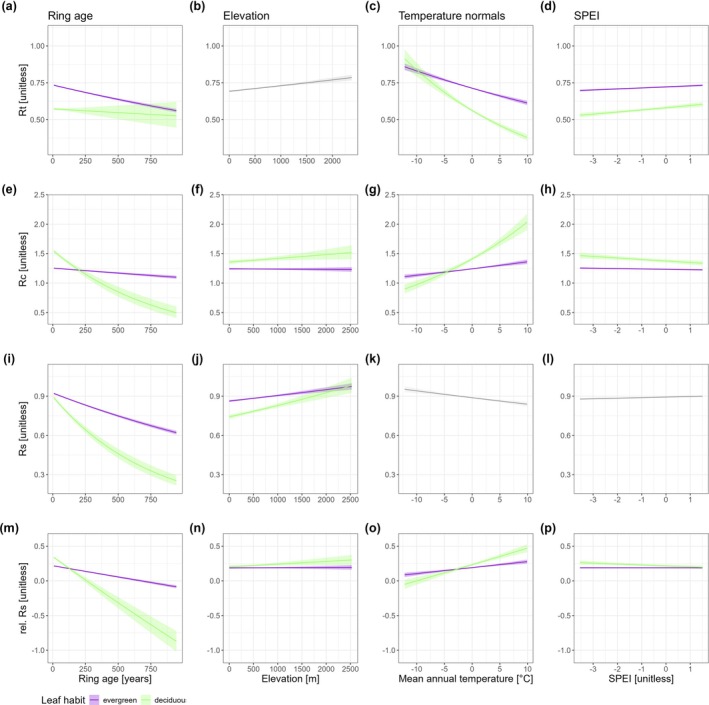
Effects of selected tree and site‐related variables with major impacts on growth resilience indices, depending on leaf habit. Variation in growth resistance, recovery, resilience, and relative resilience plotted against ring age (a, e, i, m), elevation (b, f, j, n), mean annual temperature (MAT; c, g, k, o), and the standardized precipitation evapotranspiration index (SPEI; d, h, l, p). The solid lines represent the values predicted from the linear mixed models for the evergreen (violet) and deciduous (green) species groups, with shading indicating 95% confidence intervals, obtained from the models described in Equation (2). Gray curves and shaded areas represent cases where the interaction with leaf habit was not significant (see Table 2). In these instances, leaf habit was held constant as evergreen.

The analysis of tree cumulative growth performance effects on growth resilience indices revealed distinct patterns among species. For 8 of the 23 species (35%), including all four deciduous broadleaf species, larger‐diameter trees exhibited significantly higher Rt values compared to smaller‐diameter trees (Figure S18). Conversely, Rc showed an opposite trend, with 11 of the 23 species displaying significantly lower Rc values in larger trees compared to smaller ones. Growth resilience indices varied more widely across species. Eight species showed a significantly negative relationship between Rs and tree diameter, while six species exhibited a significantly positive relationship. Rel. Rs showed a pattern similar to what was observed with Rc, 9 out of the 23 species displaying significant negative relationships. Additionally, Rt declined more sharply with ring age (tree age at the time of ring formation) in evergreens compared to deciduous species (Figure 3a). In contrast, Rs, Rc, and rel. Rs displayed opposite patterns (Figure 3e,i,m).

The temporal trends in resilience indices differed notably between deciduous and evergreen species (Table 2, Figure 2). For evergreens, no significant trends were observed in any of the four resilience indices (Figure 2d–g). In contrast, deciduous species showed significant changes from 1901 to 2017. Their Rc declined from 1.53 to 1.27, and their Rs fell from 0.86 to 0.74 (Figure 2d–f). Even after accounting for the severity of the initial drought stress, deciduous species still experienced a pronounced drop in relative resilience (Figure 2g). Notably, the Rc of deciduous species, which initially exceeded that of evergreens at the beginning of the 20th century, has converged to similar levels in recent years (Figure 2e). These changes in Rc have contributed to a significant divergence in Rs between the two groups, with deciduous species now exhibiting markedly lower Rs than evergreens (Figure 2f).

**FIGURE 4:**
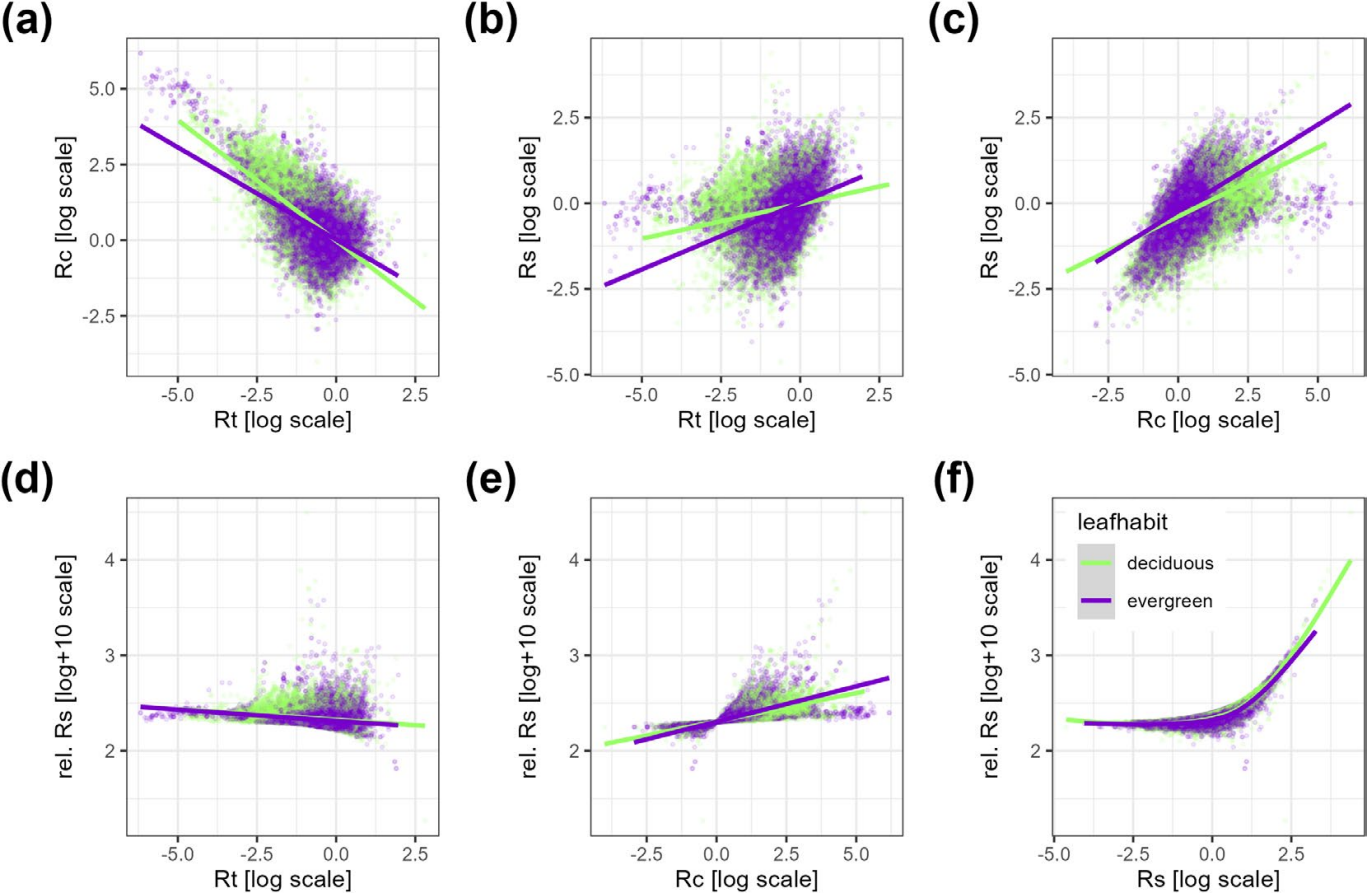
Relationships among indices of growth resilience to drought. (a) Rc against Rt, (b) Rs against Rc, (c) Rs against Rt, (d) Rt against rel. Rs, (e) Rc against rel. Rs, (f) Rs against Rel. Rs. In (a–e), lines denote linear regression lines, and in (f), lines are GAM fitting lines, added to help visual assessment of the relationships.

Our analysis revealed a negative correlation between drought resistance and recovery capacity in both evergreen and deciduous trees, with higher resistance generally corresponding to lower recovery (Figure 4a). In contrast, greater recovery was typically linked to increased resilience (Figure 4b), while the relationship between resistance and resilience was weaker (Figure 4c). These patterns remained consistent even after accounting for the initial stress amplitude, as both species groups showed a positive correlation between relative resilience and recovery, while the relationship between relative resilience and resistance remained nearly flat (Figure 4d,e). Additionally, the fitted response curve between resilience and relative resilience exhibited a slow initial increase, followed by a sharp acceleration, resembling an exponential trend in both groups (Figure 4f). Using the full resilience line as a reference, we also identified trade‐offs between resistance and recovery in both species groups (Figure S19). ANOVA results indicated that evergreens were significantly closer to achieving full resilience than deciduous species (*p* < 0.001). Deciduous species reached full resilience when resistance approached or exceeded 1, while evergreens did not. However, at very low resistance values, evergreens came closer to full resilience than deciduous species, although neither group fully recovered under these conditions.

### 3.3 Influence of Spatial Gradients on Resilience Indices

Of the three geographic variables tested, elevation had a significant and substantial influence on all the growth resilience indices (*p* < 0.001, see Table 2, Figure 3b,f,j). Both resistance and resilience to drought increased with elevation, although higher elevations were associated with slightly lower Rc values (Figure 3b,f,j). The interaction effect between leaf habit and elevation was significant for resistance (*p* < 0.05) and highly significant for resilience (*p* < 0.001; Table 2). Notably, while resilience to drought improved with elevation in both groups, this effect was more pronounced in deciduous species than in evergreens (Figure 3j). At elevations above 2200 m, the resilience of both deciduous and evergreen species approached one, indicating minimal long‐term drought impacts.

Temperature (MAT), precipitation (MAP), and drought intensity (SPEI) had highly significant effects on all the resilience indices (Table 2). Notably, the responses to temperature gradients differed between the two taxonomic groups for Rt, Rc, and relRs (Leaf habit:MAT with *p* < 0.001, Table 2), but not Rs (interaction term was nonsignificant and was not included in Table 2). As temperature increased, deciduous species exhibited a more notable decrease in Rt (Figure 3c) and a significant increase in Rc, in contrast to evergreen species (Figure 3g). Evergreens and deciduous species had significantly different responses to long‐term precipitation gradients (MAP) and drought intensity (SPEI), particularly for resistance and recovery, although not for resilience and relative resilience (Table 2, Figure 3, Figure S12). For the SPEI, which integrates both temperature and precipitation variability at an annual scale, deciduous species showed a steeper decline in Rt and a more pronounced increase in Rc as drought severity intensified (i.e., with more negative SPEI values; Figure 3d,h) than evergreens did.

### 3.4 Seasonality and Legacy Effects of Drought‐Stress Events on Resilience Indices

**FIGURE 5:**
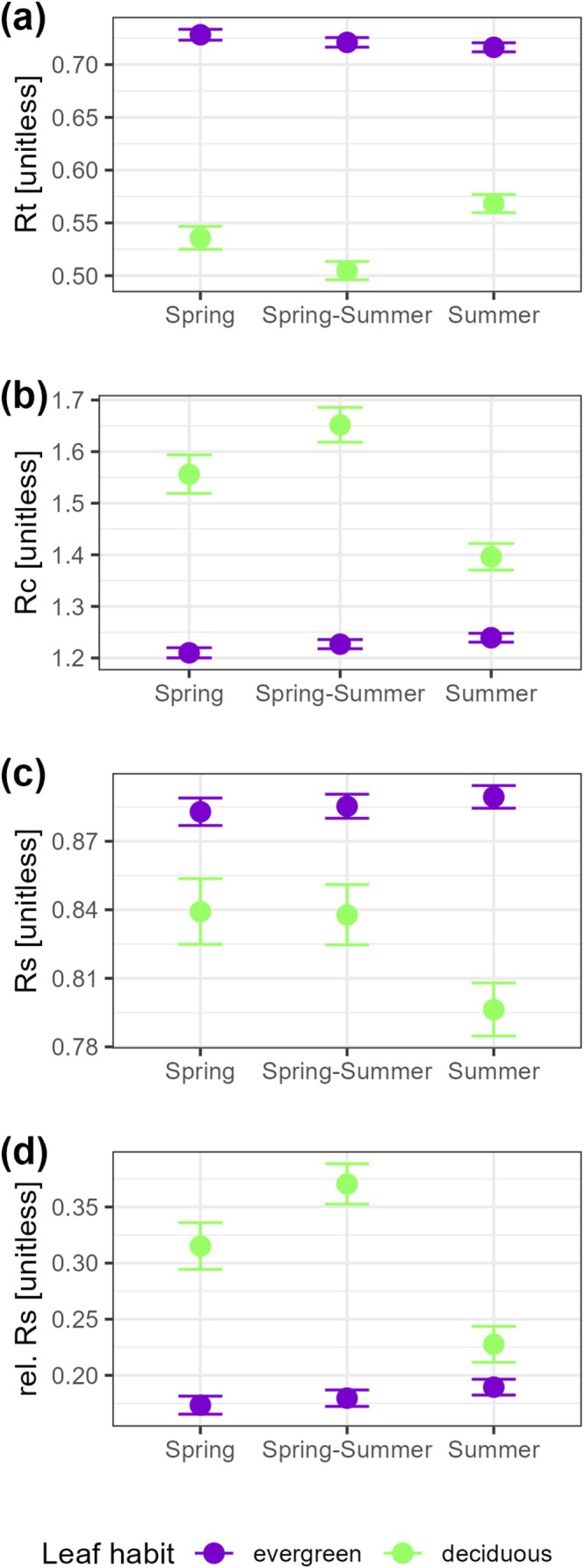
Growth resilience indices in relation to drought seasonality and leaf habits in deciduous and evergreen tree species. The predicted values of the resilience indices (a, growth resistance; b, growth recovery; c, growth resilience; d, relative resilience from the models described in Equation (2)) are presented by group, with deciduous trees in green and evergreen species in violet, considering drought‐stress events occurring in spring, summer, or both spring and summer (Spring–Summer). For each condition, the predicted values and error bars representing 95% confidence intervals are shown.

Predicted values of resilience indices depended on drought seasonality, with the notable exception of Rs values in evergreens (Figure 5, Table 2). More generally, the effect of drought seasonality was more pronounced in deciduous species, where summer droughts caused a marked decrease in Rc, Rs, and rel. Rs compared with those in evergreen trees (Figure 5).

**FIGURE 6:**
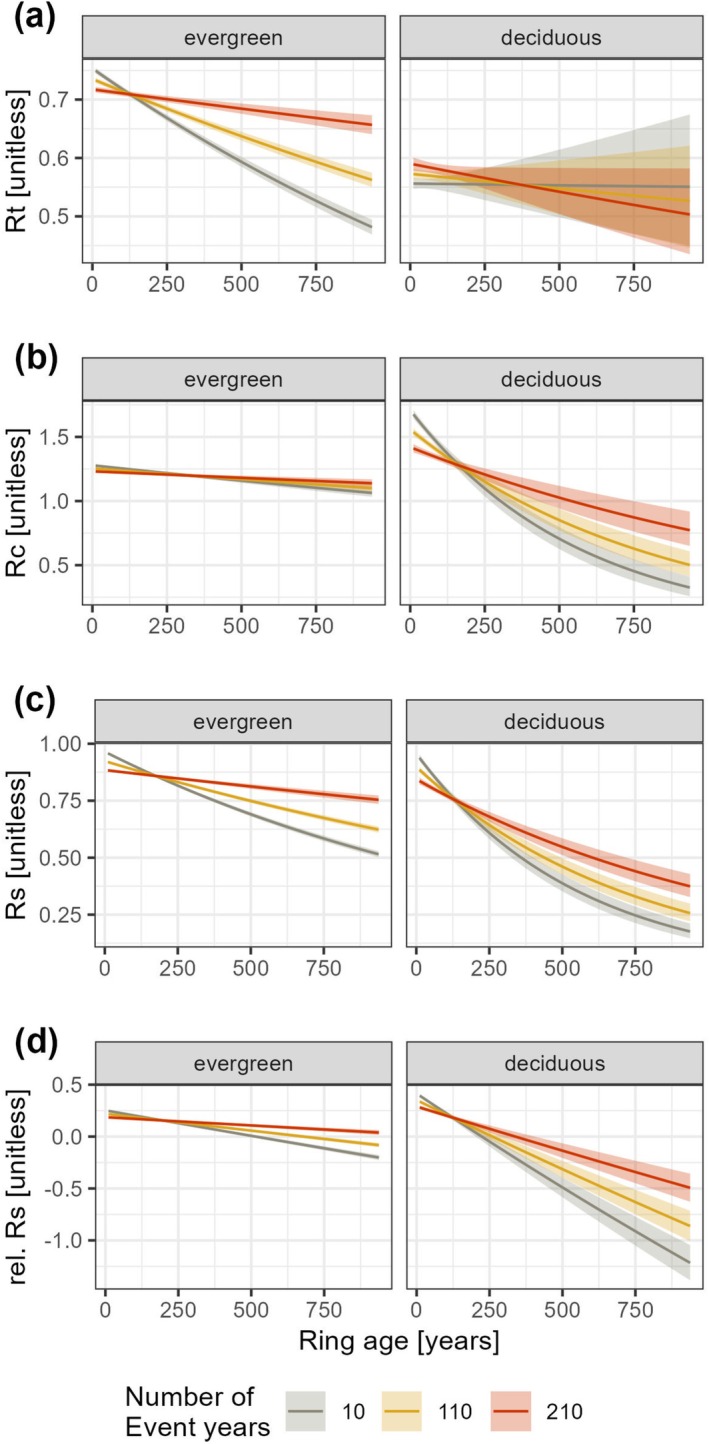
Effect of the interaction between ring age and the number of previous drought‐stress events on growth resilience indices, according to leaf habit. Growth resistance (a), recovery (b), resilience (c), and relative resilience (d) are shown for evergreen (left) and deciduous (right) species. The lines represent predicted values from linear mixed models (Equation 3; Table 2), and the shaded areas indicate 95% confidence intervals. The differently colored lines represent distinct predictions on the basis of the number of drought‐stress events considered. The gray line serves as a baseline, showing only the effect of ring age with the number of previous drought‐stress events set to zero for this model.

Previous drought exposure had a distinct and significant effect on both resistance and recovery in evergreens and deciduous trees (Table 2). Ring age had a significant negative effect on all four resilience indices (Table 2), with this impact being more pronounced in deciduous species. Specifically, the negative effect of ring age on Rc, Rs, and rel. Rs was greater for deciduous species than for evergreen species (Figure 3e,i,m). Moreover, the number of preceding drought‐stress events significantly influenced the relationship between tree ontogeny and each of the four indices, including Rs (Figure 6, Table 2). However, this effect was not observed in deciduous species, where resistance (Rt) consistently declined with increasing ring age, regardless of prior drought exposure.

Drought‐stress events occurring during a tree's early developmental stages (i.e., at lower cambial ages) with no prior drought exposure were associated with significantly higher Rc, Rs, and rel. Rs values and, in evergreens, to higher Rt values (Figure 6). However, this trend reversed once the cambial age of the wood surpassed 200 years. Beyond this point, trees that had endured multiple drought‐induced growth reductions exhibited higher Rs, rel. Rs, and Rt values than did those that had not been affected by past droughts. The influence of the number of previous drought‐stress event years on the negative relationship between ring age and Rc was also visible for deciduous species but notably less pronounced in evergreen species (Figure 6b). Note that the dataset includes 5660 trees (607 deciduous, 5053 evergreens) that reached or surpassed 200 years in age, which represents 15% of the studied trees.

## 4 Discussion

We used a very large tree‐ring dataset to investigate the drought resilience of major tree species in Canadian forests. The average Rs value of 0.9 indicates that both deciduous and evergreen species had limited resilience to drought, causing prolonged slow growth after drought occurrences. Temperature, moisture availability, and elevation were key drivers of resilience. Throughout the 20th century, resilience to drought steadily declined, particularly among deciduous trees. The historical effects of drought have differently influenced growth resistance in evergreens and recovery in deciduous species, suggesting distinct adaptation strategies to future droughts.

### 4.1 Drought Resilience Decline in Canadian Forest Tree Species

We found that 14 of 23 Canadian species had low resilience to drought events, with Rs values significantly below 1 throughout the study period from 1901 to 2017. This suggests drought can have lasting impacts on tree growth, generating prolonged growth suppression and very slow recovery to pre‐drought growth rates. Rs values were generally more likely to become significantly higher than 1 when the time‐window length was equal to or > 6 (Figure S15). This may correspond to the time required for a tree to fully recover from legacy effects of drought stress events. Such long‐term detrimental effects on growth may be caused by drought‐induced damage to a high proportion of conductive xylem, which obstructs water transport and is physiologically costly to repair (Nardini et al. 2017). Our results also revealed high variability in drought resilience among species. *Picea sitchensis* exhibited Rs values significantly higher than 1 for a time‐window length of 3 years, while *Abies lasiocarpa* , *Pinus contorta* , and *Pinus banksiana* consistently displayed values significantly below 1, regardless of the time‐window length (Figure S15). Past studies showed that tree populations from moist/rich sites in cool environments generally lack drought‐adaptive traits such as heightened stomatal responsiveness and thick cell walls, which lead to low resilience (Isaac‐Renton et al. 2018). Among widely distributed species, black spruce had slightly lower drought resilience than white spruce. While both species had resilience levels close to the evergreen average, variation in the ecological amplitudes of these two species may explain this difference. White spruce thrives in mesic sites, whereas black spruce is adapted to a broader range of environments, including xeric and hydrophilic conditions (Halliday and Brown 1943; Wirth et al. 2008). A decline in drought resilience over time in black spruce, white spruce, and several other species, including trembling aspen, paper birch, balsam fir, and jack pine, is likely driven by climate change (Figures S10 and S18). This trend is part of a broader variability in the capacity of tree species to cope with drought stress events, which appears to be shaped by trait selection in response to specific environmental conditions, e.g., different rooting habits or water‐use strategies. Tamarack, a species adapted to peatlands, exhibited the lowest resilience among all species, likely due to its dependence on high‐moisture environments that may lack drought‐adaptive traits. However, positive trends in resistance and resilience for tamarack (Figures S8 and S10) suggest that environmental changes, such as increased nutrient availability and improved soil drainage due to climate warming, could be beneficial. Among the analyzed species, red maple, known for its high plasticity (Abrams 1998), demonstrated the strongest resilience, whereas Sitka spruce, a drought‐sensitive species (W. Huang et al. 2017), ranked second‐lowest in resilience (Figure 2). These results may be partially explained by the relatively limited distribution of these two tree species.

Overall, tree growth recovery (Rc) was strongly correlated with resilience (Rs) to drought, whereas resistance (Rt) to drought was weakly associated with resilience. This suggests that resilience is more strongly driven by the capacity for post‐drought recovery, likely involving physiological adjustments and carbon dynamics, than by immediate drought resistance, which can be more constrained by external factors such as drought severity and soil water availability, in addition to species‐specific traits. This mismatch between resistance and recovery is likely a reflection of fundamental physiological, anatomical, and ecological trade‐offs in response to drought (Groover et al. 2025; Tao et al. 2024). Resistance mechanisms prioritize water conservation and hydraulic safety, often at the expense of growth. Traits such as embolism resistance (e.g., narrow, thick‐walled tracheids or vessels, thick pit membranes), osmoregulation, and hormonal control (e.g., abscisic acid regulating stomatal closure and wood adjustments) help trees maintain hydraulic function during drought. An uninterrupted hydraulic network allows trees to keep carbon assimilation at a high rate during droughts, which results in either more stable growth rates or replenished carbon reserves. In contrast, recovery emphasizes restoring growth and rebuilding hydraulic capacity, relying on stored carbohydrates and traits like efficient embolism repair and increased photosynthetic capacity.

We found that evergreens generally had higher Rt but lower Rc compared to deciduous species. Evergreens can maintain photosynthesis as long as environmental conditions permit, ensuring continuous carbon uptake, while deciduous species optimize carbon assimilation during relatively short growing seasons through traits such as enhanced osmoregulation and higher stomatal conductance (Givnish 2002; Kaproth et al. 2023; Sun et al. 2023). This contrast may explain why the impacts of drought are less severe among evergreens than among deciduous species (Rt values closer to 1; Figure 2). The additional maintenance costs of long‐lifespan needles in evergreens may also explain their slower recovery compared to deciduous trees (Song et al. 2022; Zweifel et al. 2020). Deciduous species initially exhibited high recovery, but their ability to recover from drought events gradually declined over time. By the end of the analysis period, their resilience indices were similar to those of evergreen species, with resilience remaining stable in the latter throughout the 20th century. The decline in deciduous resilience since the 1980s appears to be associated with increasing summer maximum temperatures (Figure S13a). Although summer precipitation has slightly increased during the same period (Figure S13b), it did not fully compensate for the increased atmospheric evaporative demand. As a result, summer soil moisture levels (SMI) have declined, particularly in deciduous‐dominated plots (Figure S13d), suggesting that warming‐induced moisture stress may be a key driver of reduced resilience in these stands. Studies have emphasized the increasing vulnerability of low‐resilience trees to future droughts, noting that tree mortality has been linked to rising temperatures and vapor pressure deficits (VPD) (Stovall et al. 2019; DeSoto et al. 2020). In contrast, evergreen resilience remained stable, suggesting a plateau in their ability to adapt to increasingly dry and warm conditions. Previous studies report contrasting temporal trends, with some indicating resilience increases (X. Li et al. 2020) and others showing declines (Zheng et al. 2021, 2023). Li et al. (2020) attributed resilience gains in gymnosperms to ecophysiological traits, while Zheng et al. (2021, 2023) linked resilience declines to increased sensitivity to VPD and low SPEI values. The differences between our findings and those of other studies may stem from variations in forest species composition and regional climate, as our dataset mainly represents trees from cooler and wetter regions.

### 4.2 Variation in Resilience Indices Along Environmental Gradients

Regional climate gradients significantly influenced resilience indices, with trees in warm, dry regions having lower Rt and Rs but higher Rc compared to those trees in cool, wet regions. Trees in arid climates have likely evolved mechanisms to conserve water, such as stricter stomatal regulation, which reduces carbon uptake during dry periods but protects the water‐conducting xylem tissues from long‐term damage (Chaves et al. 2016; Denham et al. 2021). In contrast, trees in humid, cool regions are adapted to maximize carbon assimilation through “looser” stomatal regulation, which increases the risk of xylem damage (Denham et al. 2021). The differences in resilience between deciduous and evergreen species were most pronounced in warm, dry regions, where deciduous species had lower resistance but higher recovery than evergreen species (Figure 3, Figure S12). In Canada, deciduous species often occupy cool, wet microsites, such as north‐facing slopes or snow accumulation areas (Burns and Honkala 1990), and tend to be more sensitive to moisture deficits due to their lack of drought adaptations (Isaac‐Renton et al. 2018). On north slopes and cool aspects, the growing season tends to be shortened. Since deciduous species already have a more limited period of activity compared with evergreens, and given their ability to shed leaves prematurely during dry periods, they may be able to recover more quickly after drought. In contrast, evergreens, maintaining year‐round physiological activity, are likely to experience more prolonged water limitations. Differences in water‐use strategies could further contribute to the observed patterns, as evergreens typically invest in conservative water use that enhances resistance but slows post‐drought recovery (Yan et al. 2024).

Resilience indices varied significantly along elevation gradients for both evergreen and deciduous species groups. Regardless of their leafing habit, trees at relatively high elevations had greater resistance and resilience to summer drought than trees at relatively low elevations. Similar trends were observed in Chinese forests (Zhang et al. 2024; Zhu et al. 2023) and at high‐altitude sites (Gazol et al. 2016), likely because of increased water input from snowmelt and high rainfall throughout the fall‐to‐spring period, which helps maintain high moisture levels. Our findings align well with the hypothesis that high‐altitude sites may act as climate refugia (Stralberg et al. 2020), where relatively cool temperatures and diverse microclimates provide protection against warming and drying trends. Whitebark pine, a high‐altitude species, had high recovery indices, likely due to its strong drought tolerance and potential benefits from warming trends that reduce temperature constraints on growth (Kichas et al. 2023). The higher levels of resilience we observed among high‐altitude trees of Canada contrast with findings from montane forests in the southwestern U.S., where resilience has declined, and mortality rates have risen (Cabon et al. 2023). Similar declines in high‐altitude trees have been observed in Mexican mountain forests (Wei et al. 2011; Sáenz‐Romero et al. 2020). These differences in high‐elevation resilience to drought reflect the warmer climate in these regions compared to Canada. Even though high‐elevation trees in Canada are currently resilient to drought, this resilience may diminish as the global climate continues to warm.

### 4.3 Understanding Drought Legacies: Ring Age and Seasonal Effects on Tree Resilience

We found negative relationships between ring age and all four drought resilience indices, aligning with previous findings that trees tend to be more resilient to drought at early life stages (Au et al. 2022; Liu et al. 2023; Lucas‐Borja, Andivia, et al. 2021; Lucas‐Borja, Bose, et al. 2021). Similarly, our models indicated that larger trees generally exhibit reduced resilience and recovery from droughts compared to smaller ones (Figure S18), which is in agreement with prior research (Camarero et al. 2024; Laverdière et al. 2022; Merlin et al. 2015). This decline may be due to higher resource demands, particularly for moisture, and the harsher environmental conditions in the upper canopy compared to the cooler, moister microclimate of subcanopy trees (Phillips et al. 2003; Campbell et al. 2021; Haesen et al. 2021).

Even when we considered pre‐ and post‐drought growth over an 8‐year time window, three of the 23 studied species failed to recover from drought stress, as indicated by mean Rs values significantly below 1 (Figure S15). This suggests that these species did not regain their pre‐stress growth performance after the initial drought impact. Incomplete recovery may stem from stress‐induced tissue damage, resource depletion, or physiological adjustments—such as shifts in carbon allocation and hormonal regulation—that constrain the tree's ability to rebuild functional xylem. Furthermore, regulatory limitations, including a reduced capacity to repair embolized vessels, may further hinder recovery (Zlobin 2024). For instance, depletion of carbohydrate reserves due to previous droughts can weaken a tree's defense mechanisms, impairing its ability to restore hydraulic function by repairing embolized xylem or forming new vessels (Schuldt et al. 2020; Anderegg et al. 2013). Persistent damage to hydraulic networks from xylem embolism can increase vulnerability to cavitation during subsequent drought events, while drought‐induced root mortality may further amplify sensitivity to water stress, compounding these effects (Anderegg et al. 2013).

The influence of ring age on resilience indices was dependent upon past experiences of drought‐related stress that caused growth reductions. Previous stress events can trigger long‐term phenotypic plasticity such as increased drought‐resistant xylem, enhanced carbon storage, or improved root growth, but often at the expense of radial growth (Hagedorn et al. 2016; J. Huang et al. 2021; Rehschuh et al. 2022; Soro, Lenz, Roussel, Larochelle, et al. 2023; Soro, Lenz, Roussel, Nadeau, et al. 2023). The accumulation of stress‐related proteins can also strengthen drought resistance (Hilker and Schmülling 2019). Our study suggests that these changes are likely most pronounced around 200 years of cambial age. Previous studies showed that older trees are generally better adapted to environmental stresses compared with younger individuals (Phillips et al. 2008; Au et al. 2022). Old trees exposed to repeated droughts may also invest more in defense mechanisms, including denser wood and thicker conduits that help reduce drought‐related damage to xylem tissues (Choat et al. 2018; Liang et al. 2021). Taken together, these factors may explain the mitigating effect of past drought exposure on the negative relationship between resilience capacity and ring age observed in this study.

In evergreens, Rt declined with age, while in deciduous species, age mainly influenced Rc. Drought timing also influenced tree responses to drought. Summer droughts reduced Rc and Rs in deciduous trees, while spring droughts had a negative impact on Rt. Deciduous species flush leaves earlier than evergreens (Swidrak et al. 2013), making trees more vulnerable to early‐season droughts. Deciduous species have narrower hydraulic safety margins than evergreens, lower leaf capacitance, and higher minimum leaf conductance, which increases the risk of xylem embolism (Blackman et al. 2019; Choat et al. 2012; Hammond and Adams 2019). Spring droughts can delay leaf unfolding, reducing carbon assimilation and leading to lower Rt in deciduous species. During summer droughts, some deciduous trees shed their leaves prematurely to conserve moisture and protect hydraulic function. While this strategy limits immediate damage, it shortens the growing season, disrupts carbon uptake, and affects growth in subsequent seasons (Descals et al. 2023; Lloret et al. 2018; Wolfe et al. 2016). High vulnerability to xylem embolism (Chuste et al. 2020; McDowell et al. 2008; Pritzkow et al. 2021) likely contributes to the strong negative effects of summer droughts on Rc and Rs. In contrast, evergreens follow a more conservative drought response strategy. Their tighter stomatal regulation, slower desiccation rates, and longer‐lived tissues enhance resistance and resilience but make them more sensitive to legacy effects from past growth conditions (Anderegg et al. 2020a; Blackman et al. 2019; DeSoto et al. 2020; Wu et al. 2022; Zweifel and Sterck 2018). Their ability to maintain carbon uptake year‐round may explain the weaker drought timing effects on resilience indices. Evergreens may also adjust to successive droughts by loosening stomatal control to sustain carbon assimilation and modifying wood anatomy to improve drought resistance (Martin‐Benito et al. 2017; Martin‐StPaul et al. 2017).

### 4.4 Assessing Tree Resilience to Drought: Challenges and Perspectives

This study is based on forest inventory data primarily obtained from dominant trees in commercially valuable stands, typically located in favorable growth sites (Duchesne et al. 2019). Because dominant trees tend to be more drought‐resilient (Au et al. 2022; Rollinson et al. 2021), our findings mainly reflect responses from the upper canopy, limiting their transferability to smaller understory trees. Moreover, trees that died from previous drought events were excluded from the analysis, potentially underestimating the full impact of drought stress on resilience (DeSoto et al. 2020). Several uncertainties must also be acknowledged. The relatively low marginal *R* ^2^ values of both species and leaf habit models indicate that the explanatory variables we tested do not capture much of the variability in resilience indices. While we did not test them in this study, genetic differences may also drive variability in resilience to drought (Depardieu et al. 2020). The absence of competition data is another limitation, as competitive interactions influence resource availability and resilience (Castagneri et al. 2022). More generally, information on neighboring trees could have allowed us to compare resilience capacities of trees growing on monospecific or mixed stands, and to include distance‐dependent competition indices in our models. However, the focus of the CFS‐TRenD database is on dendroecological analysis, and data is only available for the trees that were cored. Moreover, the absence of direct soil moisture measurements at each site may have lowered the accuracy of drought severity assessments, as soil moisture provides a more precise indication of water availability than climatic indices alone.

Despite these limitations, our study contributes to addressing key challenges in drought resilience assessments. As noted by Schwarz et al. (2020), approximately 60% of studies identify drought years based solely on climate indices. In our case, we used an approach that considered both climatic data and observed growth reductions, not to define drought years per se, but to focus our analysis on events with an observed impact on tree growth. This approach prevents misclassification of years when low SPEI values coincide with unexpected growth increases, particularly in northern or hydric forests, where reduced precipitation can alleviate excess soil moisture and enhance growth rather than induce stress (Girardin et al. 2016, 2024; Pau et al. 2021). By incorporating regional climatic variability, species‐specific responses, and potential time lags in growth decline due to stored carbon use or delayed physiological effects, our method enhances the biological relevance of drought detection. Additionally, given Canada's sparse and discontinuous weather station network, which complicates drought assessments (Ols 2017), we applied a dual‐threshold approach combining hydrological deficit levels with a modest BAI reduction (≥ 10%) to ensure that selected drought years were biologically meaningful. This approach helps distinguish actual stress events from situations where a dry spell did not significantly reduce tree growth, or even enhanced it, and provides a better account of cumulative effects on resilience. This is particularly relevant in boreal regions, which represent most of our study area, where trees growing in waterlogged soils for much of the year may benefit from reduced water input.

Our findings also emphasize the importance of selecting an appropriate time window for calculating resilience indices, as this choice significantly influences results and interpretation (Figure S15, Tables S3–S6). Since physiological data are not always available to determine the optimal time window for each species, we recommend systematically assessing this parameter when applying Lloret resilience indices. Given the variability in species traits and drought characteristics, careful time window selection is crucial for ensuring robust results. Finally, while our study relies on the growth‐based indices proposed by Lloret et al. (2011), resilience assessments would benefit from a multi‐proxy approach integrating ecological, physiological, and environmental data (Albrich et al. 2020; Nikinmaa et al. 2020). Future research should incorporate remote sensing, soil moisture data, and additional physiological indicators to provide a more comprehensive understanding of forest responses to climate extremes and disturbances.

## 5 Conclusions

Our findings indicate that both deciduous and evergreen species had low drought resilience overall, with deciduous species experiencing a significant decline in recovery capacity, now reaching levels similar to the historically low resilience of evergreens. This suggests that current growth conditions are increasingly deviating from the ecological optima of deciduous species. The existence of “drought legacies” and their effects on drought resilience underscore the contrasting adaptation strategies between evergreen and deciduous species. The low drought resilience capacities we observed suggest that Canadian forests may become less productive with more frequent and severe droughts, posing risks to wood resource availability and a variety of other forest ecosystem services. The negative trends in deciduous species resilience we observed suggest that significant changes in forest composition may occur in the future, either leading to a reduction in deciduous stands or to a shift toward stands dominated by deciduous species currently found in southern, warmer regions. Despite these challenges, our study highlights the capacity of both deciduous and evergreen species for some degree of adaptation to past droughts, suggesting potential resilience to future climate stressors. Our findings emphasize an urgent need for proactive forest management and long‐term planning to safeguard Canadian forest productivity and biodiversity as the climate changes.

## Author Contributions

**William Marchand:** conceptualization, data curation, formal analysis, investigation, methodology, visualization, writing – original draft. **Claire Depardieu:** conceptualization, data curation, formal analysis, investigation, methodology, supervision, validation, visualization, writing – review and editing. **Elizabeth M. Campbell:** investigation, supervision, writing – review and editing. **Jean Bousquet:** investigation, supervision, writing – review and editing. **Martin P. Girardin:** conceptualization, data curation, funding acquisition, investigation, methodology, project administration, resources, supervision, validation, writing – review and editing.

## Conflicts of Interest

The authors declare no conflicts of interest.

## Supporting information


Data S1.

## Data Availability

The data that support the findings of this study are openly available in Figshare at https://doi.org/10.6084/m9.figshare.29377538. Raw tree‐ring measurements were obtained from the Natural Resources Canada TreeSource repository and are available upon request at https://treesource.rncan.gc.ca/en/treerings. Additional tree‐ring data were obtained from the International Tree‐Ring Data Bank (ITRDB) from the NOAA Paleoclimatology repository at https://www.ncdc.noaa.gov/data‐access/paleoclimatology‐data/datasets/tree‐ring. Weather data were obtained from the Environment and Climate Change Canada's Historical Climate Data portal at https://climate.weather.gc.ca/ and BioSIM server at https://cfs.nrcan.gc.ca/projects/133.
