# Supplementary material for: Long‐Term Temporal Divergence in Post‐Drought Resilience Decline Between Deciduous and Evergreen Tree Species
Source: Glob Chang Biol. 2025 Jul 8;31(7):e70330. doi: 10.1111/gcb.70330 (PMC12235587; doi:10.1111/gcb.70330)
Supplement: Supplementary file 1 — Data S1. [file GCB-31-e70330-s001.docx]

**Supplementary Information for “Long-term temporal divergence in post-drought resilience decline between deciduous and evergreen tree species**

William Marchand, Claire Depardieu, Elizabeth M. Campbell, Jean Bousquet, Martin P. Girardin

This document contains 20 figures and 7 tables

**List of supplementary figures:**

- Figure S1. Sample depth (number of tree rings) measured per year and species.

- Figure S2. Age distribution of the sampled trees, by species.

- Figure S3. Diameter at breast height (DBH) distribution of the sampled trees, by species.

- Figure S4. Investigation into the potential impacts of first-order autocorrelation on the detection of drought events and the inclusion of trees in analyses.

- Figure S5. Diagnostic plots from linear mixed models fitted for growth resistance (Rs), recovery (Rc), resilience (Rs), and relative resilience (rel. Rs).

- Figure S6. Spatial variation in tree growth sensitivity to soil moisture deficit in Canada.

- Figure S7. Mean order-1 autocorrelation function by species.

- Figure S8. 95% confidence intervals of the predicted values of Rt against calendar year, by species.

- Figure S9. 95% confidence intervals of the predicted values of Rc against calendar year, by species.

- Figure S10. 95% confidence intervals of the predicted values of Rs against calendar year, by species.

- Figure S11. 95% confidence intervals of the predicted values of Rel. Rs against calendar year, by species.

- Figure S12. Relations between growth resilience indices and total annual precipitation normals (1981-2010).

- Figure S13. Temporal trends in summer (June-August) maximum temperature, total precipitation, Standardized Precipitation Evapotranspiration Index (SPEI), and Soil Moisture Index (SMI) over the period 1901-2019.

- Figure S14. Frequency (A) and proportion (B) of years characterized by a significant reduction in growth rates by drought conditions, as identified with the SPEI and both reductions in growth rates and drought.

- Figure S15. Resilience indices by species and length of time window.

- Figure S16. Distribution maps of deciduous species.

- Figure S17. Distribution maps of evergreen species.

- Figure S18. Relations between resilience indices and lifetime growth performance.

- Figure S19. Comparisons of the relationships between observed values of recovery and resistance to the line of full resilience.

- Figure S20. Correlation matrix of continuous explanatory variables used in the leaf habit models

**List of Supplementary Tables:**

- Table S1. Effect sizes from Cohen’s d tests comparing the distribution of first-order autocorrelation between trees that were either retained or excluded from analyses for each tested time window.

- Table S2. Models’ terms retained in analyses, scientific justification of their inclusion, and relevant references.

- Table S3. Results from growth resistance (Rt) models considering individual time windows, ranging from 2 to 8 years.

- Table S4. Results from growth recovery (Rc) models considering individual time windows, ranging from 2 to 8 years.

- Table S5. Results from growth resilience (Rs) models considering individual time windows, ranging from 2 to 8 years.

- Table S6. Results from relative resilience (rel. Rs) models considering individual time windows, ranging from 2 to 8 years.

- Table S7. Outputs from the species linear mixed models (Eq. 2 in the main document), for each of the four resilience indices.

**List of Supplementary Methods:**

- Methods S1: Assessing the impact of autocorrelation on tree selection and drought detection.
- Methods S2: Assessing resilience indices in relation to long-term tree growth


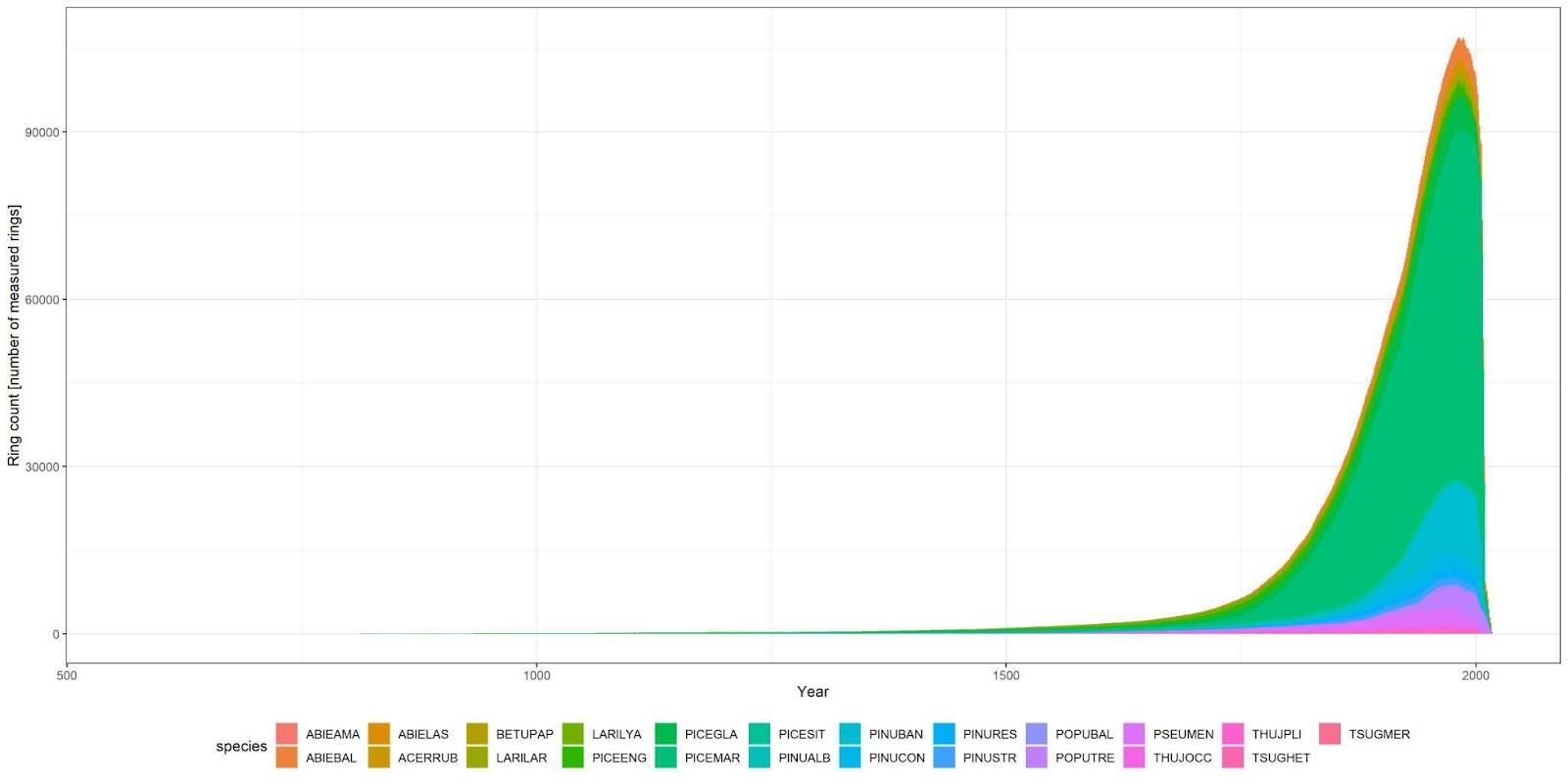


**Figure S1. Sample depth (number of tree rings) measured per year and species.**


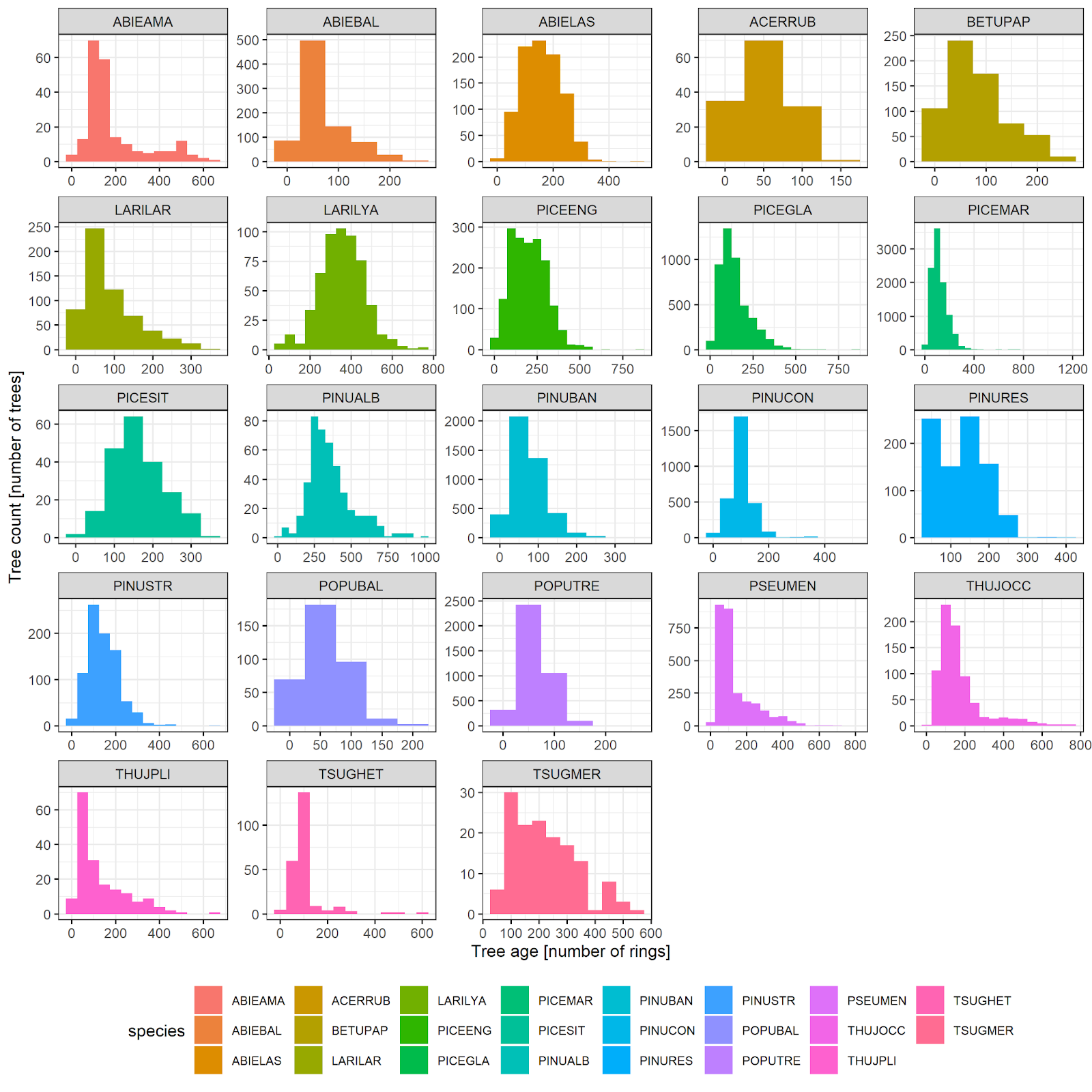


**Figure S2. Age distribution of the sampled trees by species.** Tree age was computed as the number of years between the first and last recorded years, representing the tree’s biological age. This approach ensures that the age reflects the tree's entire lifespan rather than just a portion of its growth period.


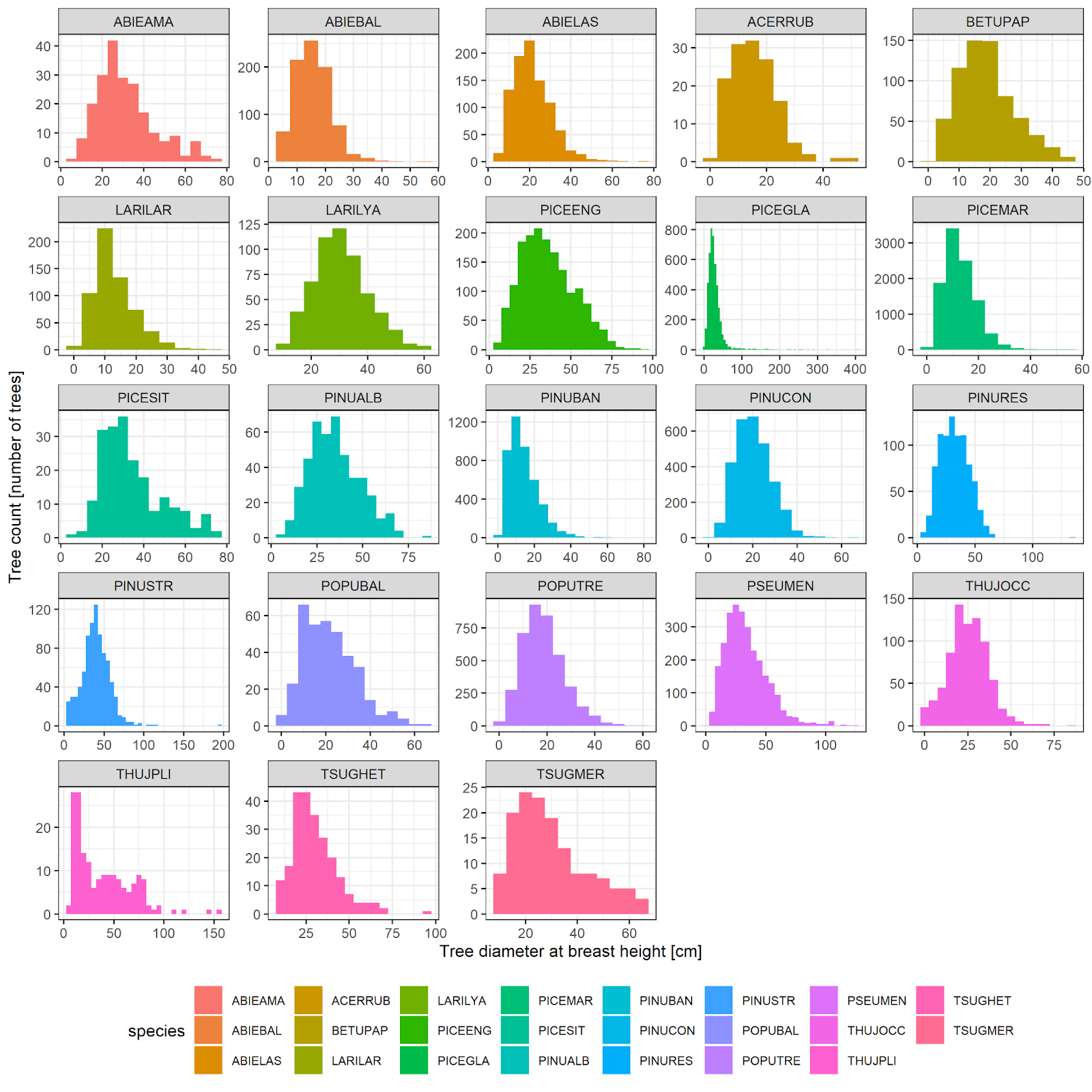


**Figure S3. Distribution of the diameter at breast height (DBH) of the sampled trees by species.** The DBH was computed as two times the sum of the radial growth increments recorded.


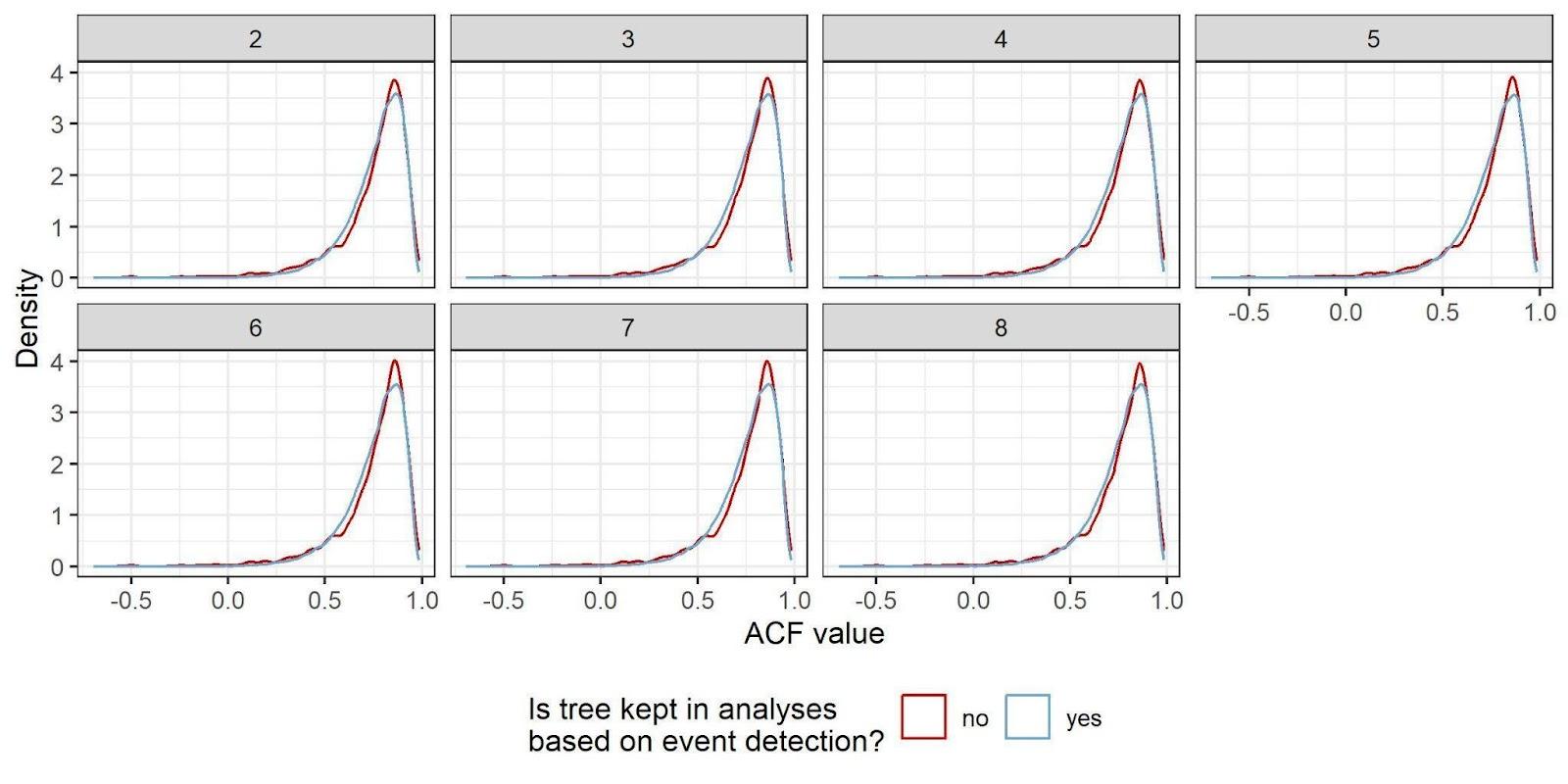


**Figure S4. Investigation into the potential impacts of first-order autocorrelation on the detection of drought events and the inclusion of trees in analyses.** Density plots of tree-level first-order autocorrelation function values for each tested time window (from 2 to 8), separated on the basis of whether trees were included or excluded from our analyses owing to the detection of a drought event.


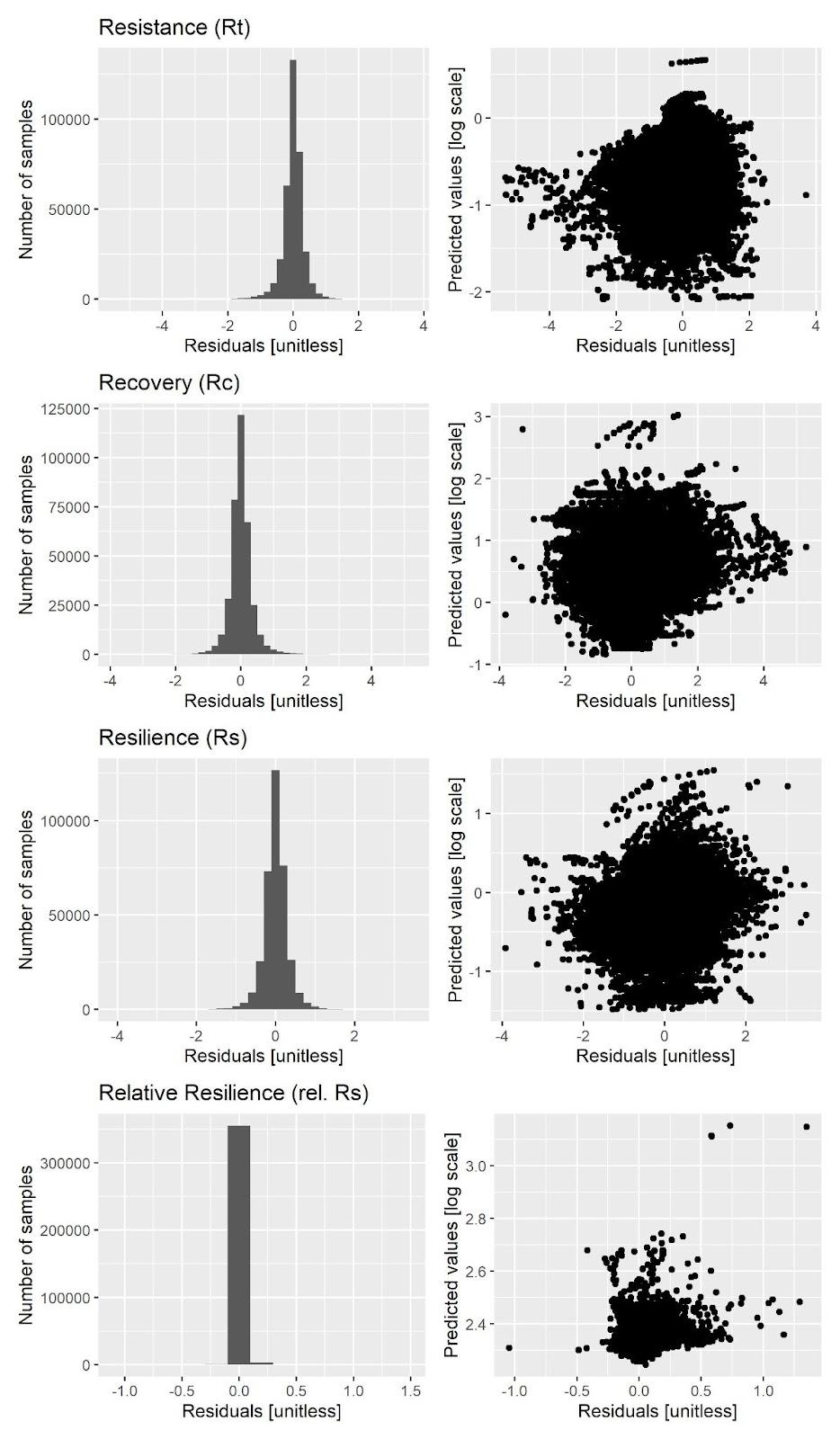


**Figure S5. Diagnostic plots from linear mixed models fitted for growth resistance (Rs), recovery (Rc), resilience (Rs), and relative resilience (rel. Rs).** For each resilience index, the left side displays the distribution of residuals used to assess the normality assumption, while the right side shows the scatterplot of residuals against the predicted values used to assess the homoscedasticity assumption.


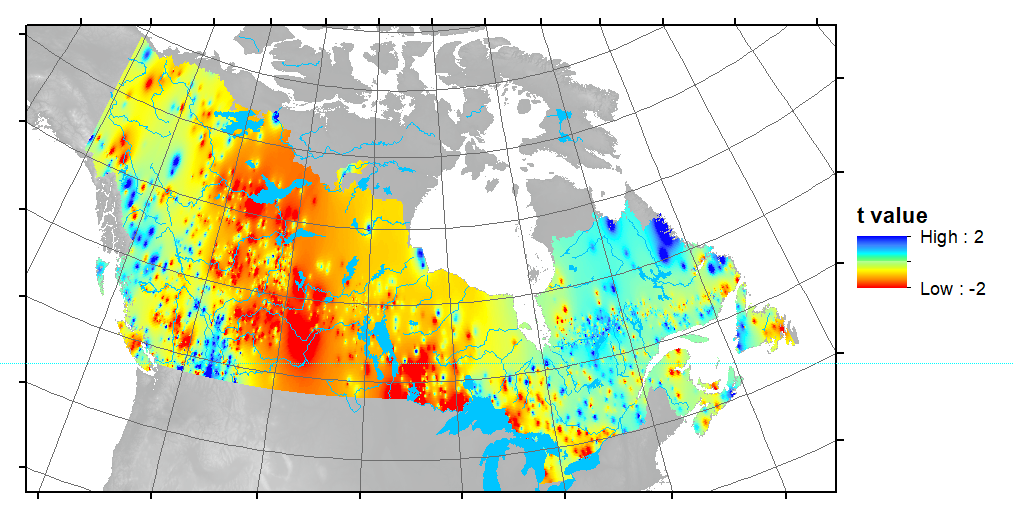


**Figure S6. Spatial variation in tree growth sensitivity to soil moisture deficit in Canada.** The map represents the strength of relationships (t-values) between tree basal area increments and the illustrated factor for the period of 1951–2018 as examined using site-level generalized additive mixed models (for details please see Girardin et al. 2024). Red indicates areas where high values of the specified variable have a negative impact on tree growth, and blue indicates areas where high values favor tree growth.


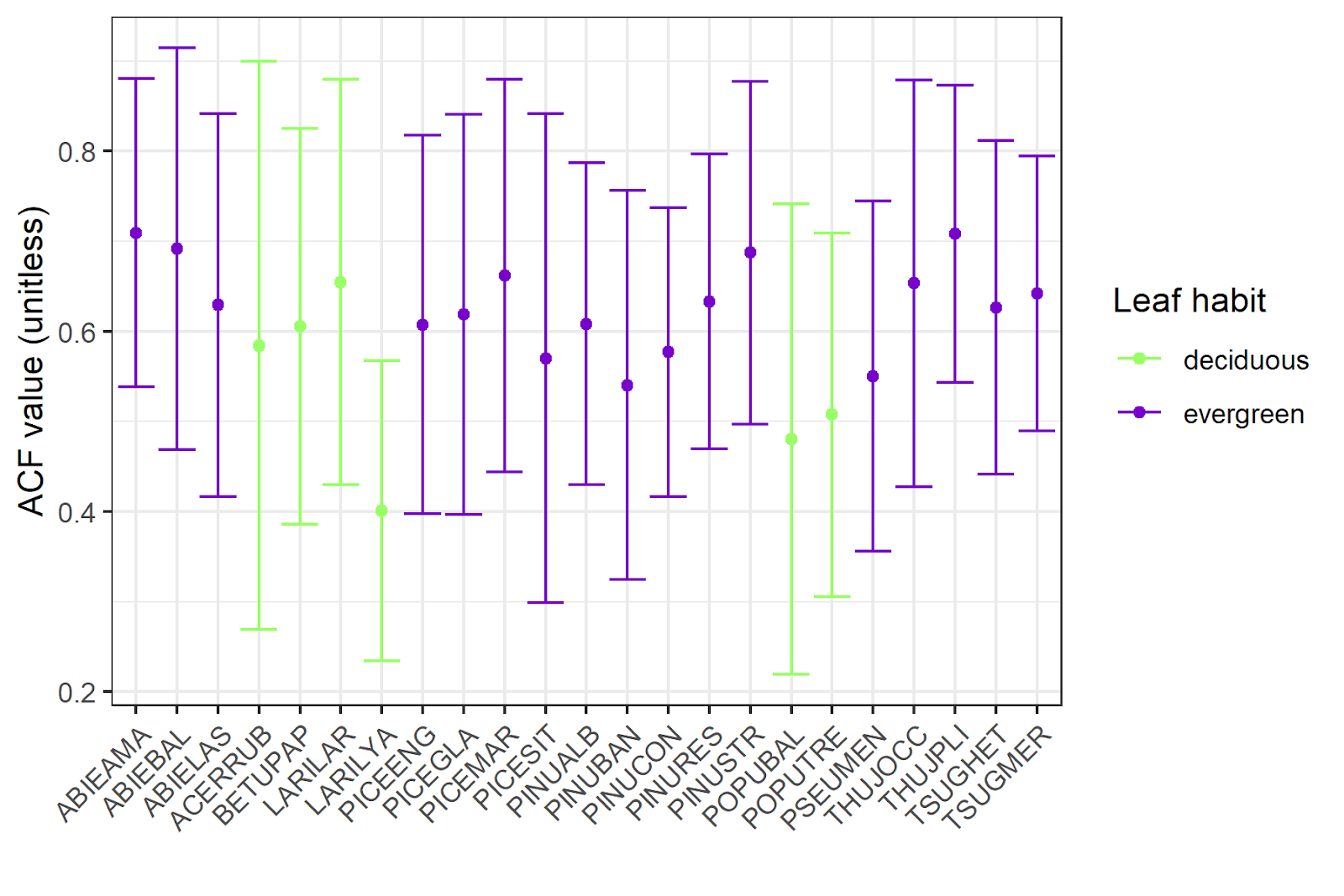


**Figure S7. Mean order-1 autocorrelation function by species.** Error bars are standard deviations from the means.


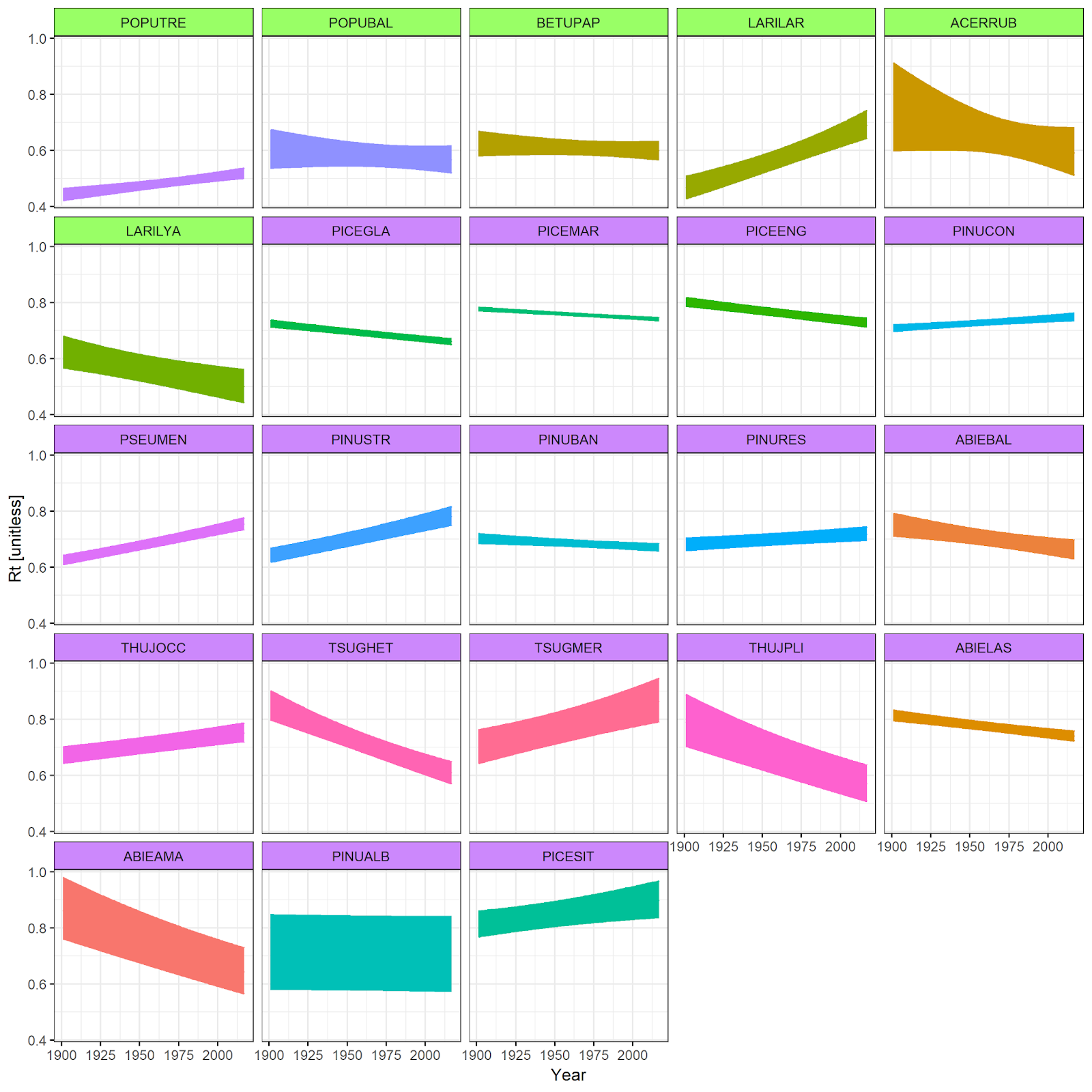


**Figure S8. 95% confidence intervals of the predicted values of Rt against calendar year, by species.** Confidence intervals were obtained from species-level linear mixed models including Year, Ring Age and Tree Size as explanatory variables, and time window length, timing of growth decline and plot identity as nested random effects.


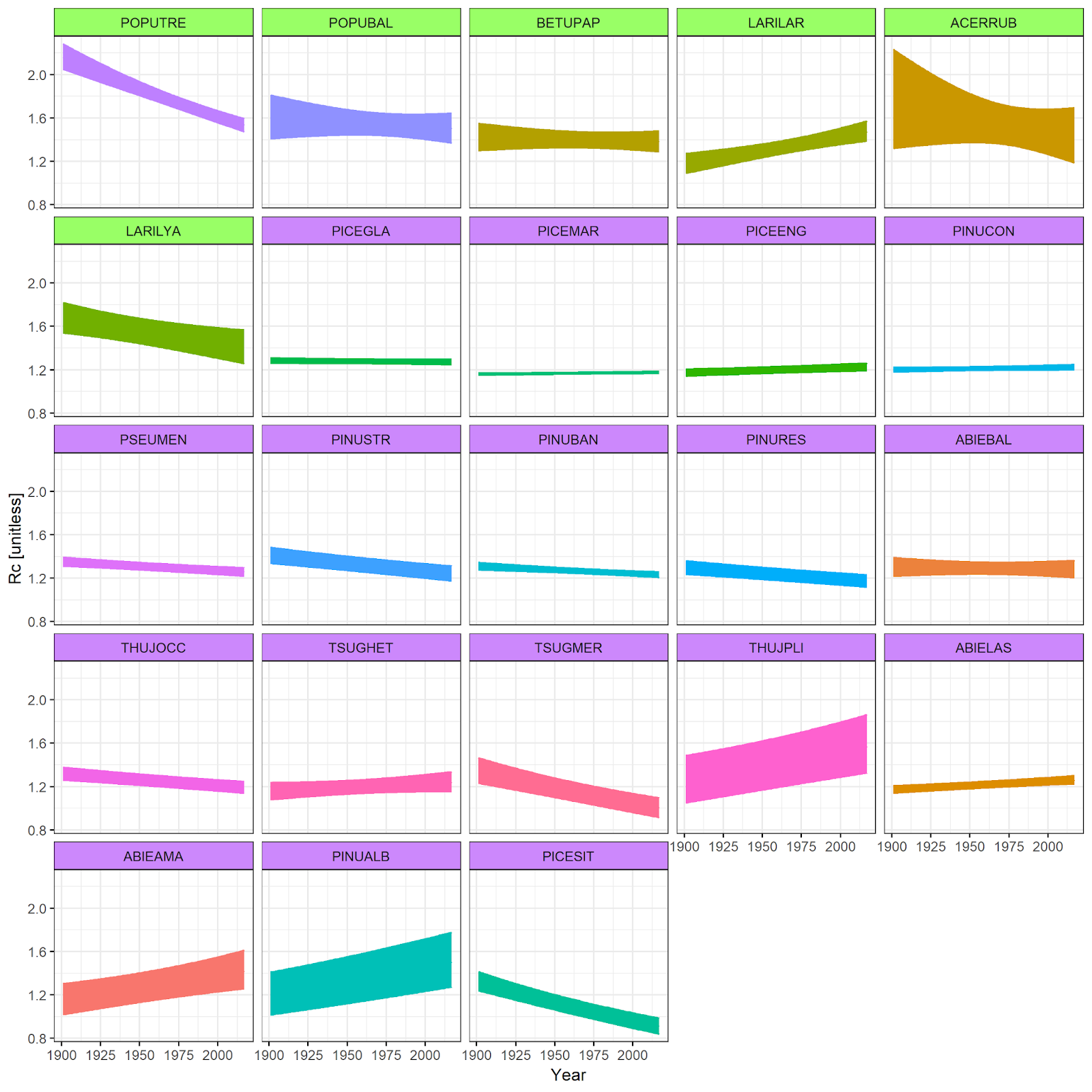


**Figure S9. 95% confidence intervals of the predicted values of Rc against calendar year, by species.** Confidence intervals were obtained from species-level linear mixed models including Year, Ring Age and Tree Size as explanatory variables, and time window length, timing of growth decline and plot identity as nested random effects.


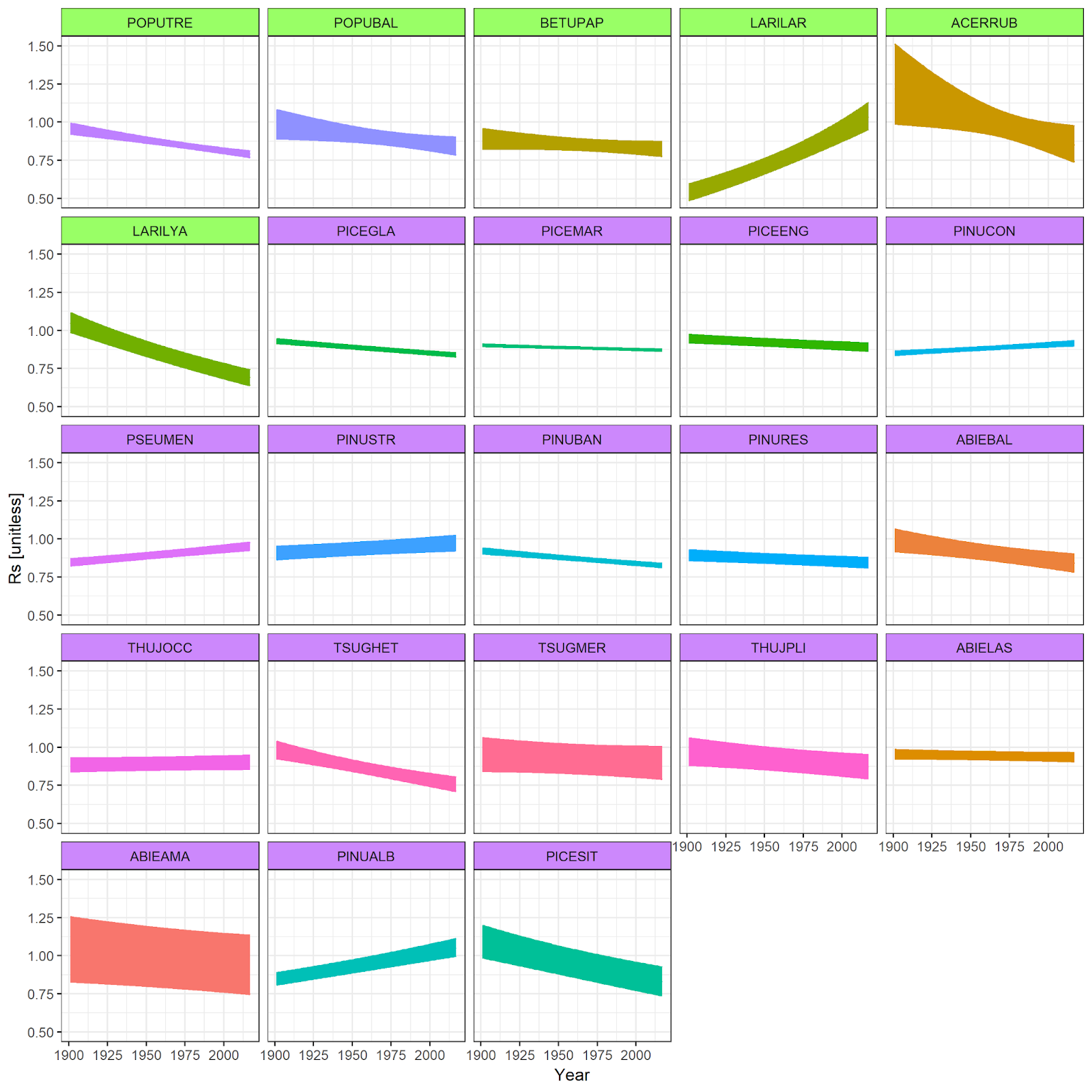


**Figure S10. 95% confidence intervals of the predicted values of Rs against calendar year, by species.** Confidence intervals were obtained from species-level linear mixed models including Year, Ring Age and Tree Size as explanatory variables, and time window length, timing of growth decline and plot identity as nested random effects.


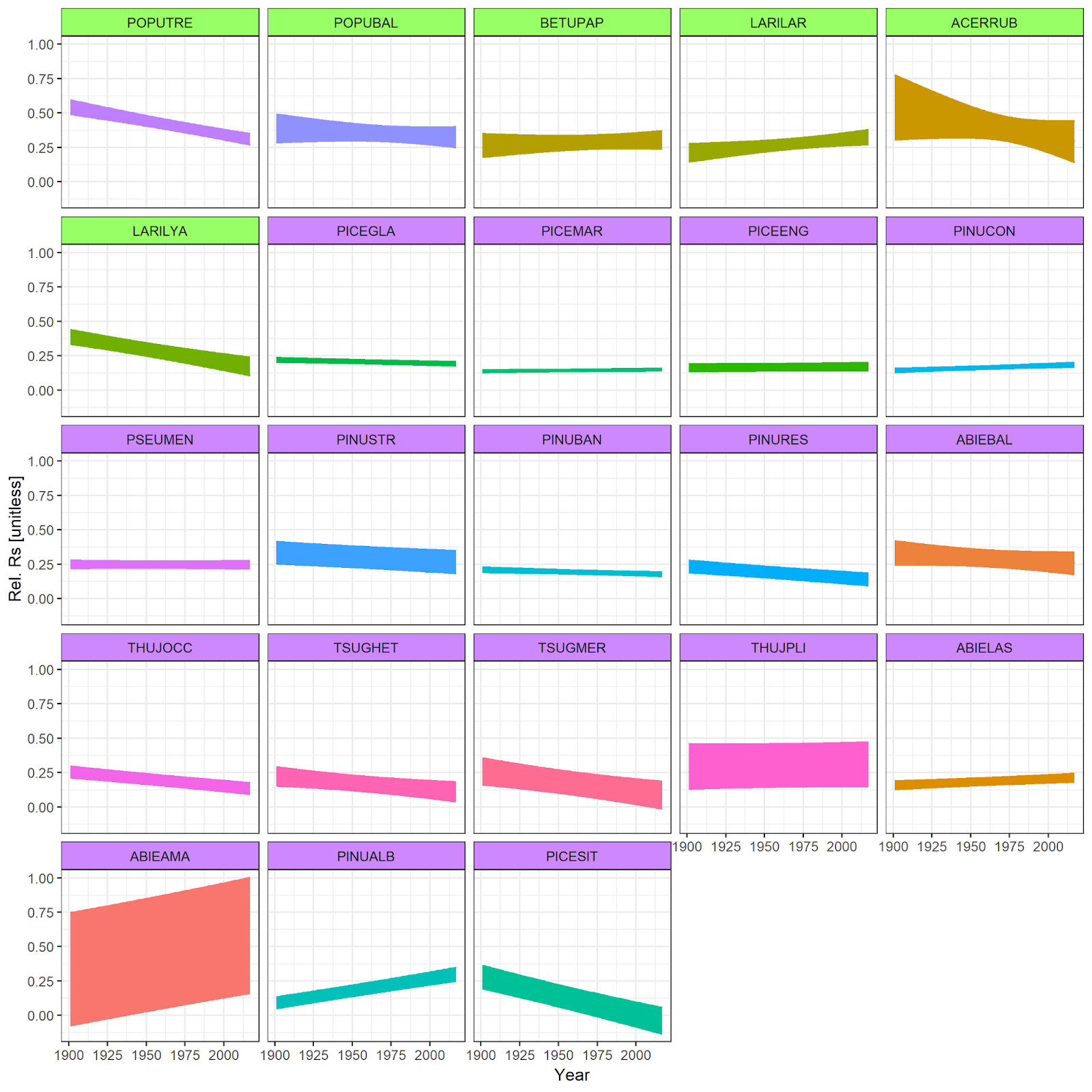


**Figure S11. 95% confidence intervals of the predicted values of Rel. Rs against calendar year, by species.** Confidence intervals were obtained from species-level linear mixed models including Year, Ring Age and Tree Size as explanatory variables, and time window length, timing of growth decline and plot identity as nested random effects.


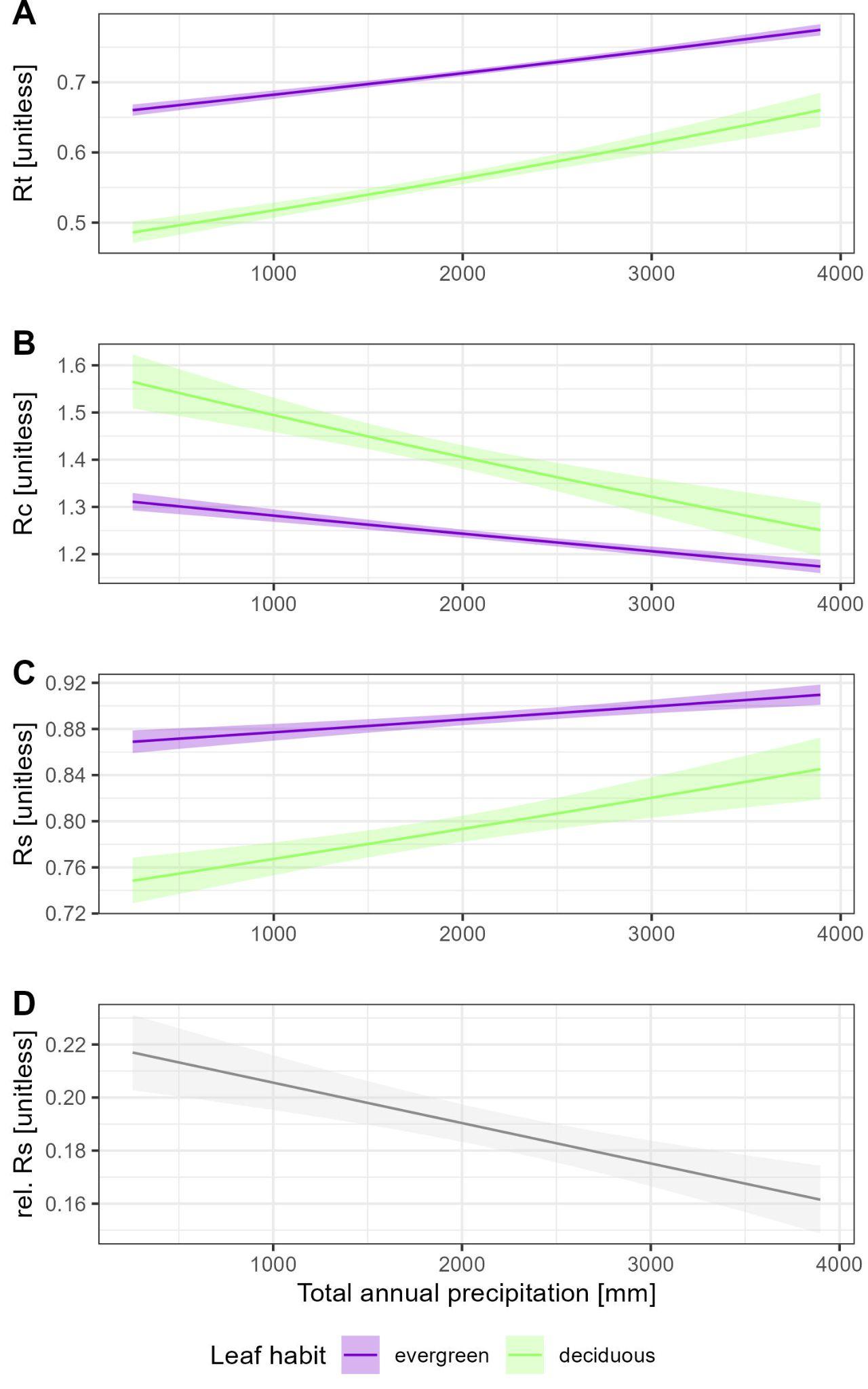


**Figure S12. Relations between growth resilience indices and total annual precipitation normals (1981-2010).** The predicted values obtained from the models depicted in Eq. 2 in the main document (straight lines) are displayed together with 95% confidence intervals (shadings). In panel (D), the gray line and shading depict the average effect of the precipitation gradient on relative resilience, as the interaction between MAP and leaf habit was not significant and therefore excluded from the final model.


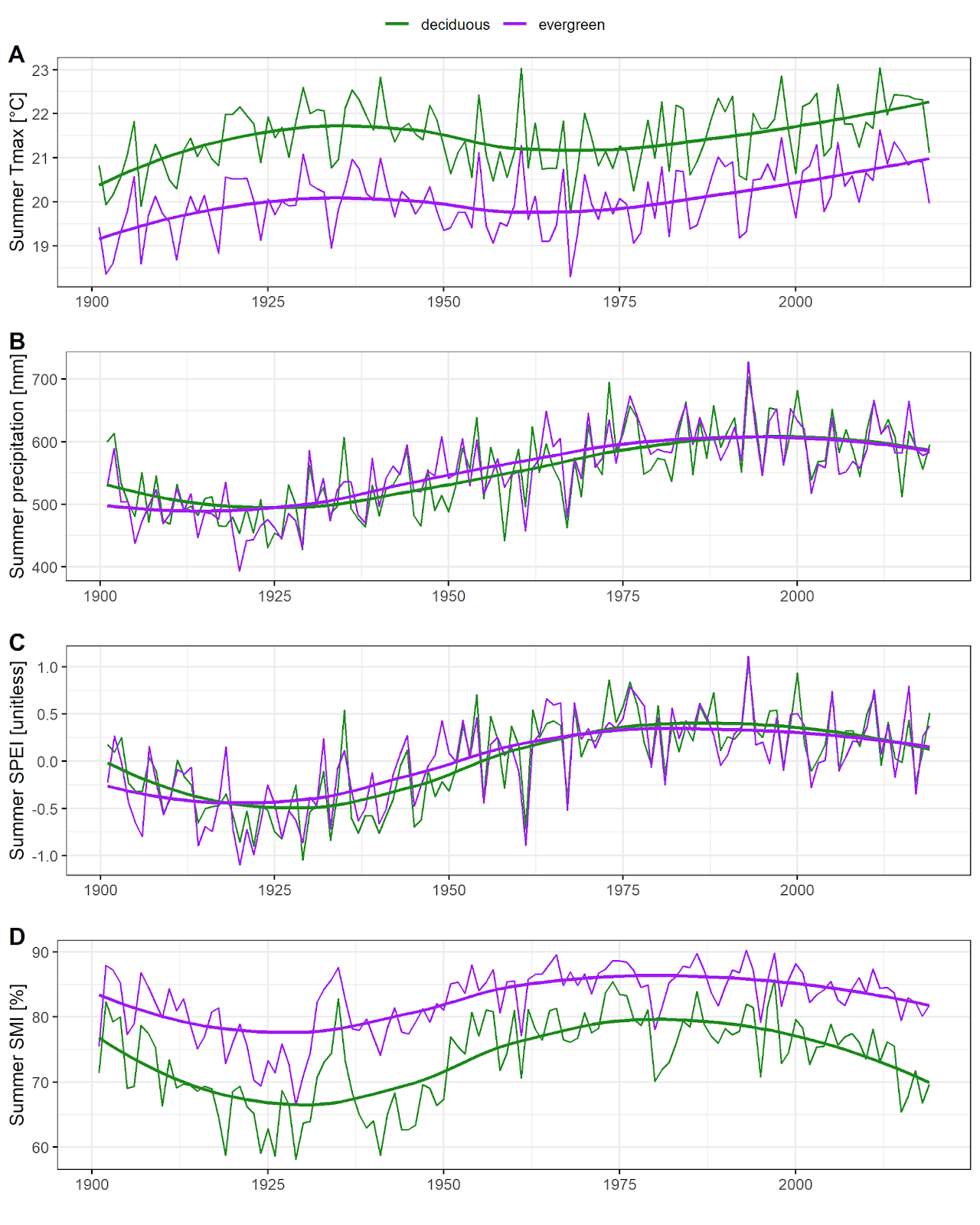


**Figure S13. Temporal trends in the summer (June-August) maximum temperature, total precipitation, Standardized Precipitation Evapotranspiration Index (SPEI), and Soil Moisture Index (SMI) over the period 1901-2019.** For each climatic variable, the temporal variations are shown separately for plot locations with deciduous versus evergreen species, along with LOESS smooth lines. The climate data were obtained from BioSIM 11.


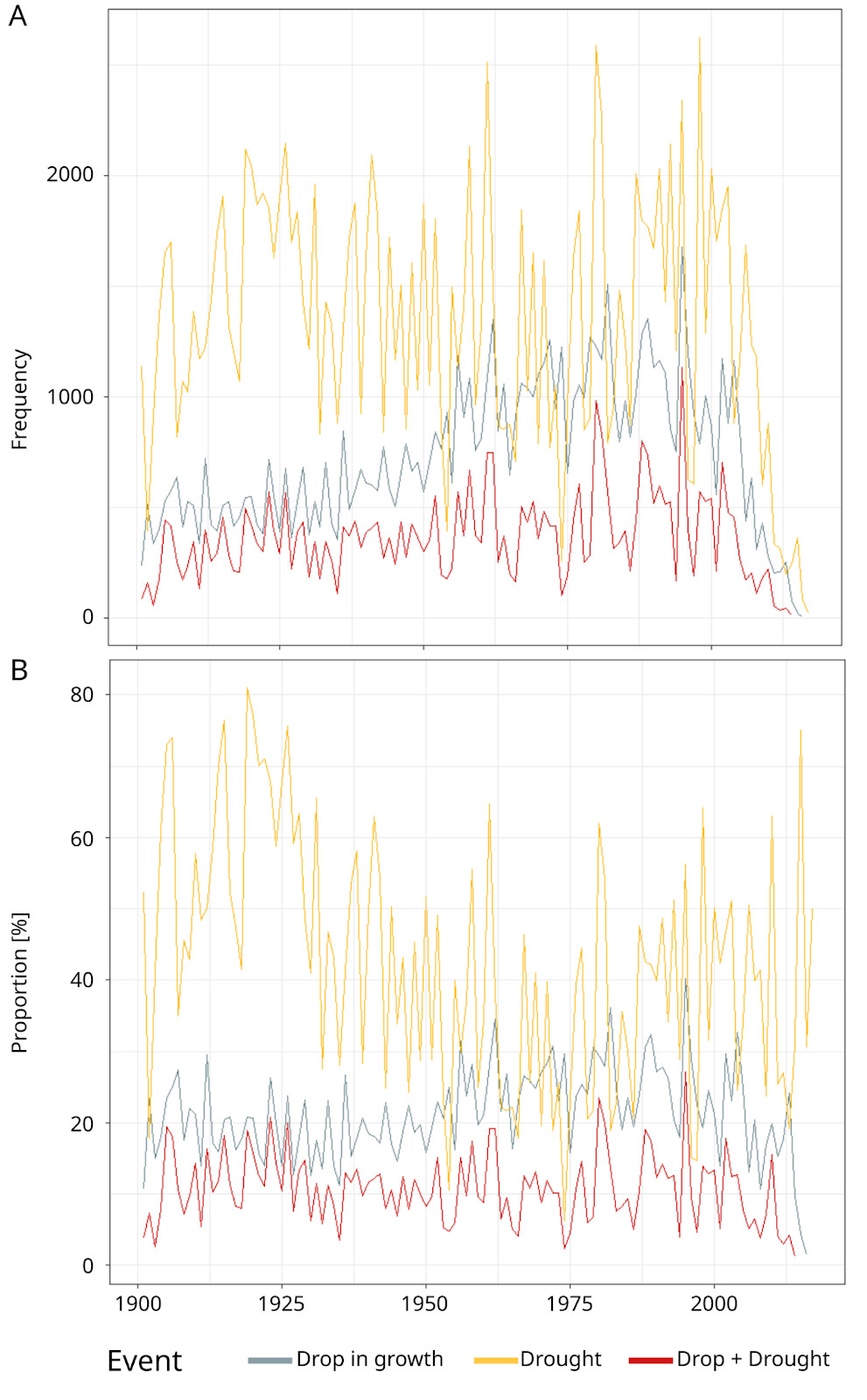


**Figure S14. Frequency (A) and proportion (B) of years characterized by a significant reduction in growth rates, by drought conditions, as identified with the SPEI and both reductions in growth rates and drought.**


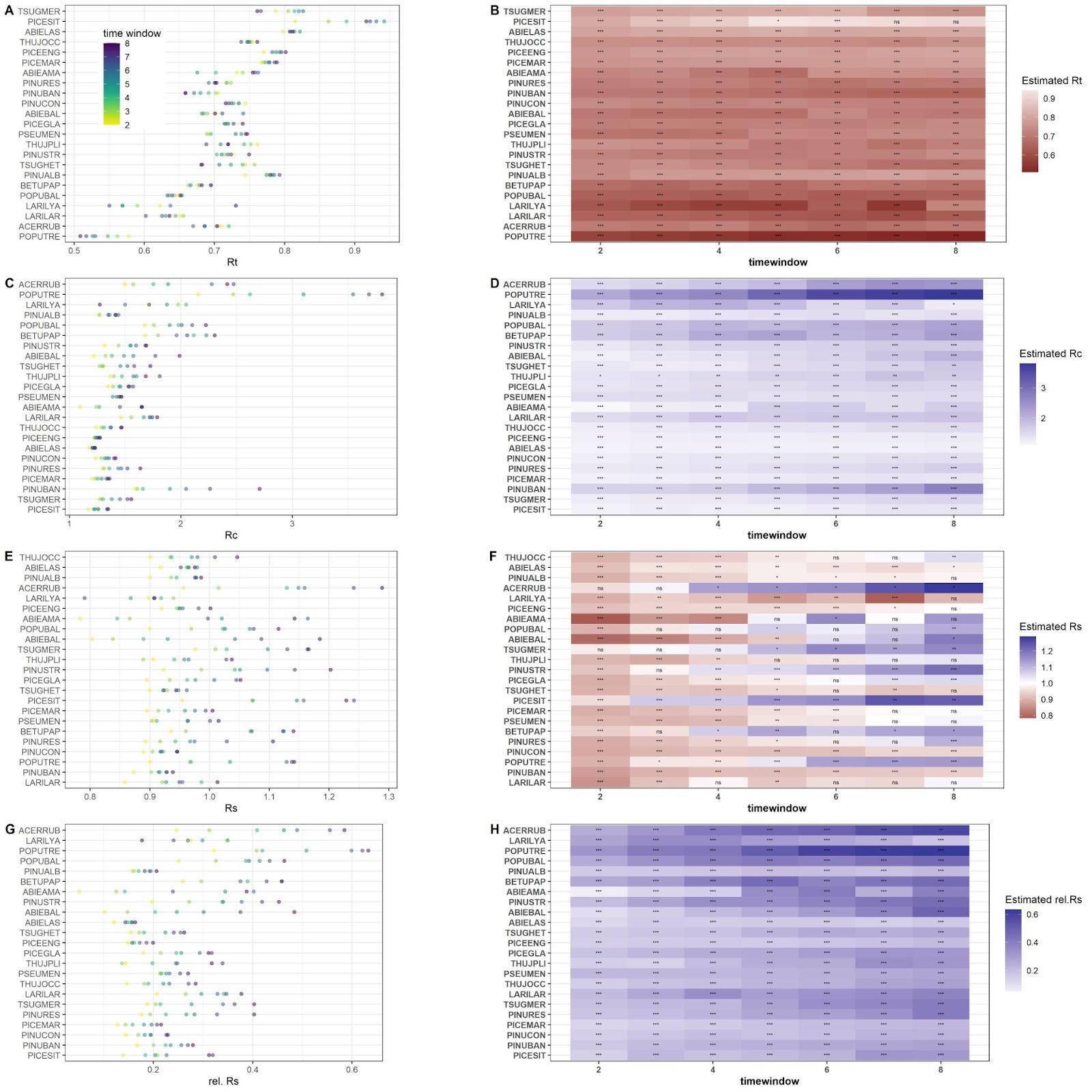


**Figure S15. Resilience indices by species and length of time window.** Left panels (A, C, E, G) display arithmetic means of resilience indices, by time window and species. Right panels are results from t-tests evaluating if the resilience indices are significantly different from one, by species and time window. “***” = *P* < 0.001, “**” = *P* < 0.01, “*” = p-value < 0.05 and “ns” denotes values not significantly different from 1 at *P* = 0.05.


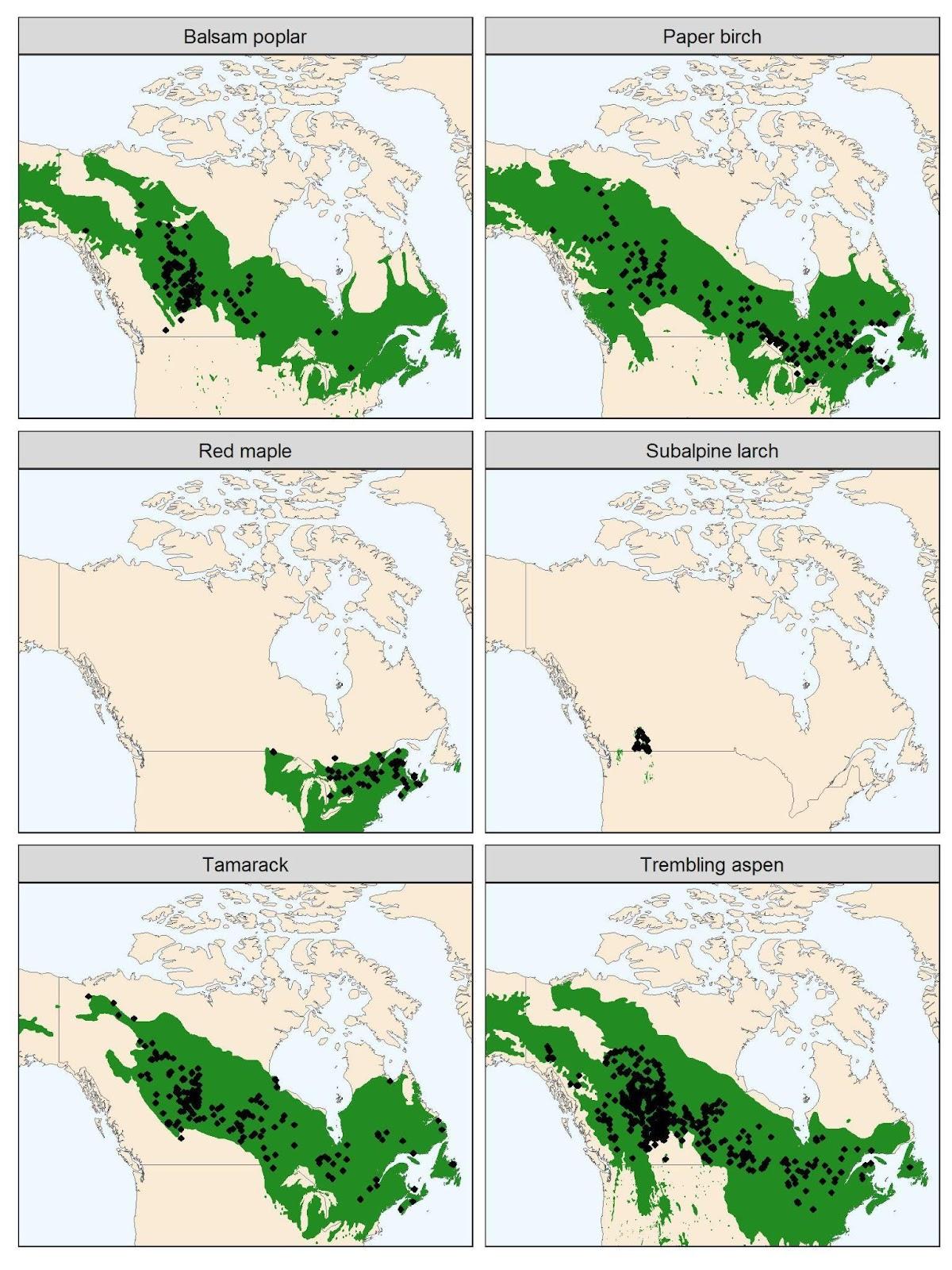


**Figure S16. Distribution maps of deciduous species**. Green areas denote natural distribution areas of species, and black diamonds show sample plot locations. Shapefiles of species distribution areas are based on Little’s range maps, and were downloaded from the [USGS website](https://web.archive.org/web/20170127093428/https:/gec.cr.usgs.gov/data/little/). Reference: Little, E. L. 1971. Atlas of United States Trees, Volume 1, Conifers and Important Hardwoods. Washington, DC: US Department of Agriculture Miscellaneous Publication.


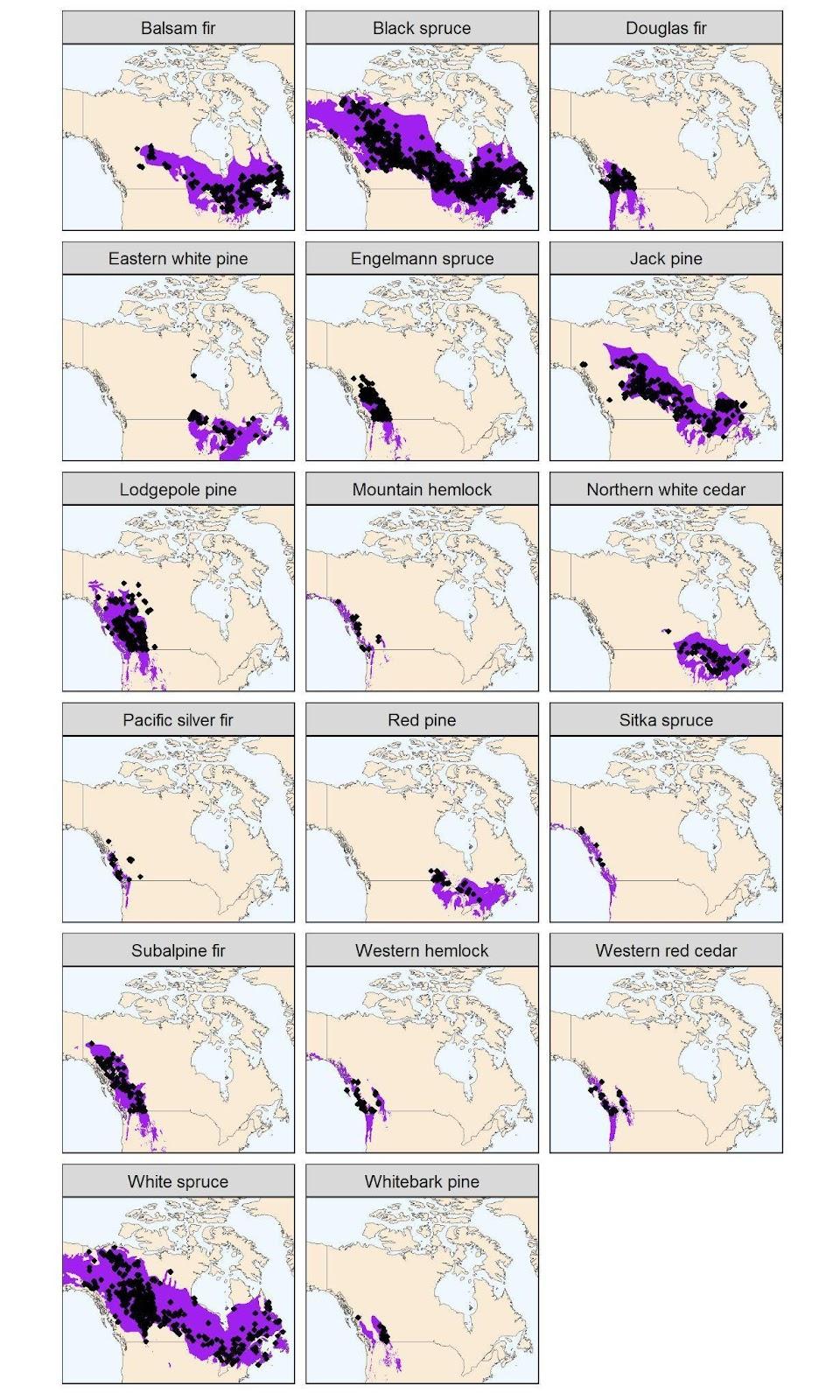


**Figure S17. Distribution maps of evergreen species**. Purple areas denote natural distribution areas of species, and black diamonds show sample plot locations. Shapefiles of species distribution areas are based on Little’s range maps, and were downloaded from the [USGS website](https://web.archive.org/web/20170127093428/https:/gec.cr.usgs.gov/data/little/). Reference: Little, E. L. 1971. Atlas of United States Trees, Volume 1, Conifers and Important Hardwoods. Washington, DC: US Department of Agriculture Miscellaneous Publication.


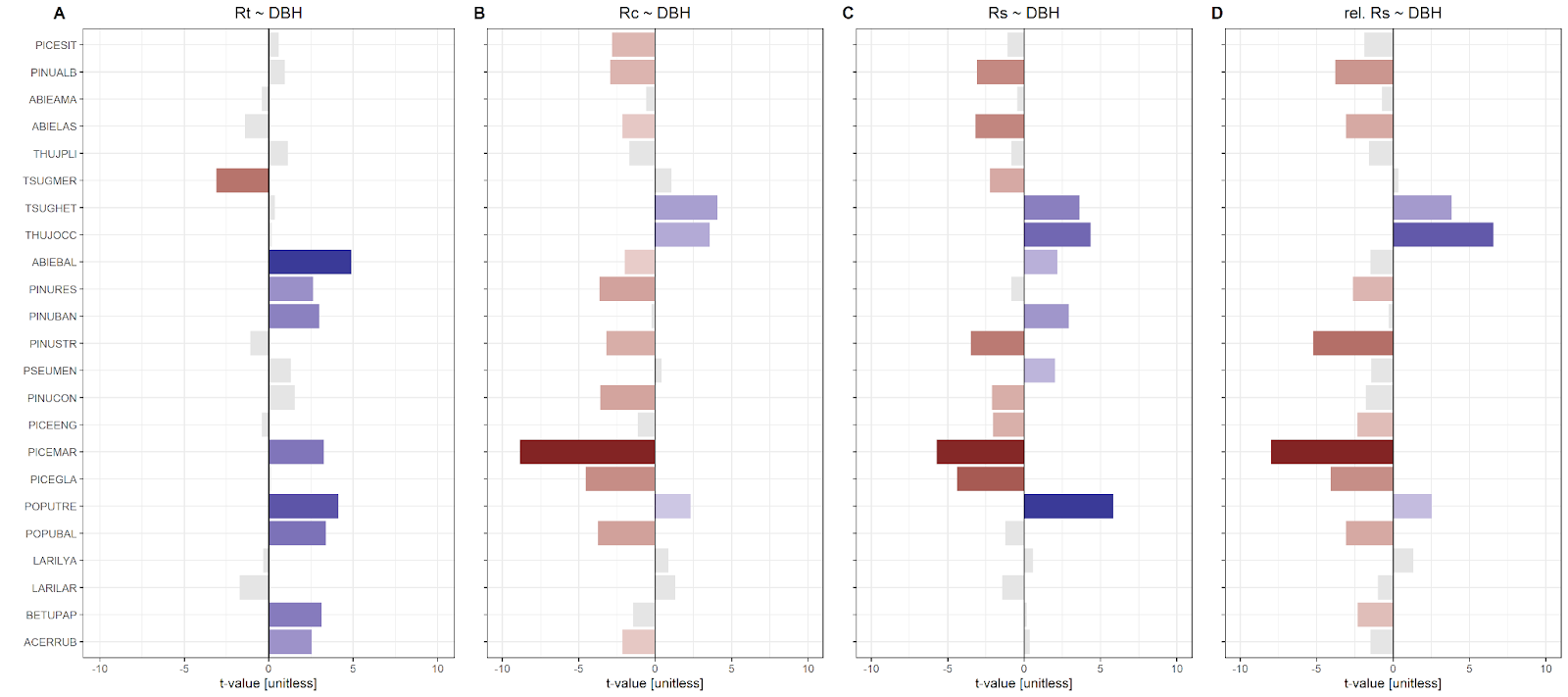


**Figure S18. Relations between resilience indices and lifetime growth performance**. Linear mixed models were fitted by species, considering each of the three resilience indices averaged at the tree level as the response variable and tree diameter at breast height (DBH) as the explanatory variable. 69 models were fitted in total (23 species × three resilience indices). The time window length and plot identity were added as random effect factors. The response variables were log-transformed to ensure the homoscedasticity of the residuals. t-values are reported by species. The gray bars represent nonsignificant results at ɑ = 0.05. The color gradient indicates the strength and direction of the relationship with red shading representing increasingly negative trends and blue shading representing increasingly positive trends. Gray-colored bars display non-significant values. The models were fitted using the function *lme* of the r-package *nlme* (Pinheiro and Bates, 2019).

**Figure S19. Comparisons of the relationships between observed values of recovery and resistance (purple and green lines) to the line of full resilience (red line)**
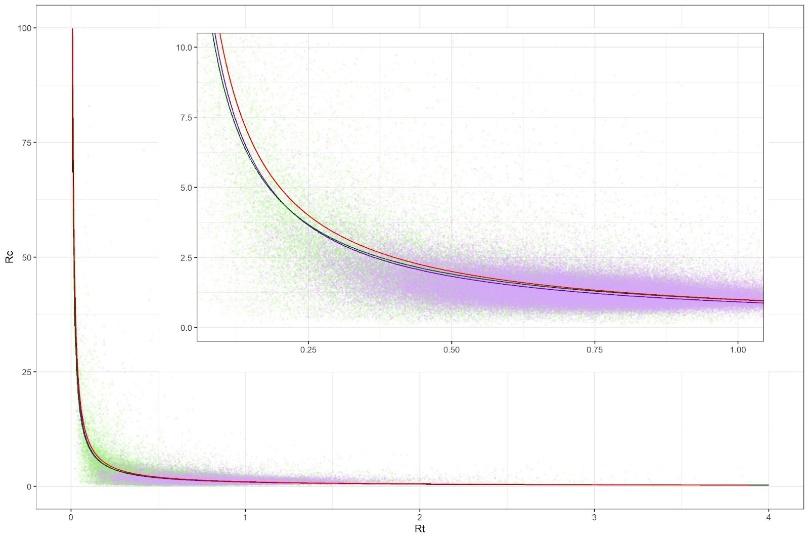
, which represents a hypothetical function where growth recovery is equal to the inverse of resistance. Negative exponential curves were fitted to the observed data separately for evergreen and deciduous species, with the following equations: Rc = 0.9911*Rt^-0.9451 for deciduous species and Rc = 0.9143*Rt^-0.9977 for evergreen species. The fit of the real data for deciduous (green line) and evergreen (purple line) trees was significantly better than the fit to the data using the hypothetical line of full resilience (in red, P < 0.001***) for the two species groups. This was determined by comparing residuals using t-tests.


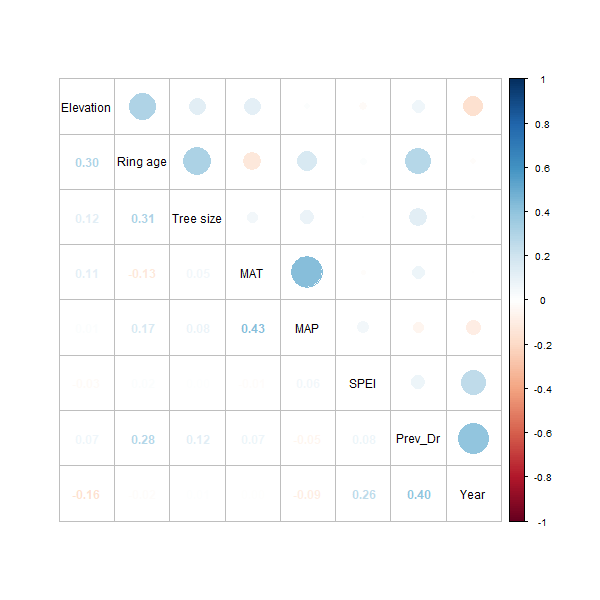


**Figure S20. Correlation matrix of continuous explanatory variables used in the leaf habit models** (as described in Eq. 3 in the main document).

**Table S1. Effect sizes from Cohen’s d tests comparing the distribution of first-order autocorrelation between trees that were either retained or excluded from analyses for each tested time window.**

| Cohen’s d | Time window |
| --- | --- |
| -0.076 | 2 |
| -0.078 | 3 |
| -0.078 | 4 |
| -0.074 | 5 |
| -0.052 | 6 |
| -0.058 | 7 |
| -0.054 | 8 |

**Table S2. Models’ terms retained in the species and leaf habit models (see “Model” column), scientific justification of their inclusion, and relevant references. The full references listed in this table are provided at the end of this Word document.**

| Type | Term | Model | type | Explanation | Reference |
| --- | --- | --- | --- | --- | --- |
| single term | leaf habit | leaf habit models | categorical | Differences in life history traits can lead to differences in drought resilience capacities | Kaproth et al. 2023 |
|  | tree age | species models + leaf habit models | continuous | In trees, ageing leads to a weakened xylem network and changes in carbon allocation strategies that could influence drought resilience capacities | Lucas-Borja et al, 2021 |
|  | tree size | species models + leaf habit models | continuous | Higher moisture and carbon needs for metabolism can lead to lower resilience capacities in bigger trees compared to small-sized individuals | Trugman et al. 2018 |
|  | past drought exposure | leaf habit models | continuous | Cumulative effects of droughts, e.g. on the proportion of embolized vessels or on carbohydrate reserves can exacerbate growth reduction over time | Gazol et al, 2020 |
|  | drought season | leaf habit models | categorical | Different tree developmental phases are impacted depending on the seasonality of drought. Spring droughts affect leaf development, leading to reduced photosynthetic capacity early in the season. Summer droughts deplete carbohydrate reserves, hindering the tree’s ability to recover. | Bose et al. 2021 |
|  | SPEI | leaf habit models | continuous | SPEI accounts for differences in drought intensity. More intense droughts (more negative SPEI values) generally have more detrimental effects on tree growth rates, | Bottero et al, 2021 |
|  | MAT | leaf habit models | continuous | MAT accounts for regional temperature gradients. Some regions display warm climate, while others display cool climate. These variations can influence the selection of life history traits and, consequently, the drought resilience capacities of tree populations. | Isaac-Renton et al, 2018 ; Serra-Maluquer et al, 2022 |
|  | MAP | leaf habit models | continuous | MAP accounts for regional gradients in precipitation. Tree populations in regions with ample precipitation inputs could lack adaptations to moisture stresses | Serra-Maluquer et al, 2022 |
|  | elevation | leaf habit models | continuous | Soil parameters, such as texture and depth, vary along the altitudinal gradient, as do slope aspect, CO2 concentration, wind intensity, and snow accumulation. All these factors can influence the drought resilience capacities of trees. | Jiang et al., 2024; Stralberg et al., 2020 |
|  | year | leaf habit models | continuous | Accounts for the temporal trend in drought resilience capacities of trees. | Li et al., 2020; Zheng et al., 2021, 2023 |
|  | species ID | species models | categorical | Different species exhibit distinct trait sets, which contribute to varying drought resilience capacities. | Jiang et al, 2024 |
| interaction terms | leaf habit x tree age | leaf habit models | interaction | The expected negative effect of tree age on resilience capacities could be stronger in deciduous than in evergreen species due to their higher initial vulnerability to drought-induced embolism and the higher carbon demand required to replace leaves seasonally. | Song et al. 2022; Hasselquist et al. 2010; Ruehr et al. 2019 |
|  | tree age x past drought exposure | leaf habit models | interaction | In younger trees, the cumulative negative effect of past drought exposure on drought resilience may be less severe due to higher plasticity. In older trees, the effects can be more severe, or they may be mitigated by a 'stress memory.' | Groover et al. 2024; Rodriguez-Zaccaro & Groover 2019; Gessler et al. 2020 |
|  | leaf habit x drought season | leaf habit models | interaction | Deciduous and evergreen species exhibit different phenologies: deciduous trees grow new leaves each season to photosynthesize, while evergreen trees retain their leaves year-round, allowing for continuous photosynthesis. Summer leaf shedding in some deciduous species, often linked to drought stress, can reduce their resilience to drought. | Li et al. 2023 |
|  | leaf habit x SPEI | leaf habit models | interaction | Different water use and drought response strategies exist between deciduous and evergreen species: the long-lived leaves of evergreens may make them more sensitive to severe and prolonged droughts, while deciduous species can shed their leaves to mitigate the impacts of severe drought episodes. | Song et al. 2022; Vargas et al. 2021 |
|  | leaf habit x MAT | leaf habit models | interaction | Regional temperature gradients have different effects on the drought resilience of deciduous versus evergreen species due to variations in trait selection. | Kaproth et al. 2023 |
|  | leaf habit x MAP | leaf habit models | interaction | Regional precipitation gradients have different effects on the drought resilience of deciduous versus evergreen species due to variations in trait selection. | Kaproth et al. 2023 |
|  | past drought exposure x leaf habit | leaf habit models | interaction | Depending on the set of functional traits, the rates of damage accumulation may differ, and the stress memory effect could be absent in one of the two groups. | this study |
|  | leaf habit x elevation | leaf habit models | interaction | Deciduous and evergreen species possess different traits that make them adapted to high-altitude conditions in distinct ways. Evergreens may be better suited to these environments, as they are ready to photosynthesize as soon as conditions become favorable, despite the short growing season. | Jiang et al, 2024 |
|  | past drought exposure x tree age x leafhabit | leaf habit models | interaction | The modulating effect of tree age on resilience capacities can differ between evergreen and deciduous species due to their distinct sets of functional traits. | this study |
|  | past drought exposure x elevation | leaf habit models | interaction | Different rates of stress memory and accumulation of embolized vessels may occur depending on the position along the elevation gradient. | this study |
|  | leaf habit x year | leaf habit models | interaction | Trajectories in drought resilience indices can differ between leaf habit groups due to different adaptation strategies. | Li et al., 2020; Zheng et al., 2021, 2023, this study |
| random effects | plot ID | species models + leaf habit models | categorical | A random effect of plot ID is included to account for site variability (uncontrolled variables) and the non-independence of samples (as multiple trees are sampled from the same plot). | e.g. Stone et al. 2011 |
|  | timing of growth decline | species models + leaf habit models | categorical | Accounts for the legacy effects of drought | Bose et al, 2024 |
|  | time window length | species models + leaf habit models | categorical | Accounts for differences inherent to the pre and post-drought period lengths | Schwarz et al. 2020 |
|  | species ID | leaf habit models | categorical | Different species exhibit distinct trait sets, which contribute to varying drought resilience capacities. | Jiang et al, 2024 |

**Table S3.** **Results from growth resistance (Rt) models considering individual time windows, ranging from 2 to 8 years**. T-values and significance levels are displayed (*** = *P* < 0.001; ** = *P* < 0.01; * = *P* < 0.05, ‘ns’ = nonsignificant effect). Positive t-values with significant effects are highlighted in green, negative t-values with significant effects are highlighted in red, and nonsignificant effects are highlighted in gray. For comparison purposes, the exact same list of variables and interactions as in the main models were retained, even if nonsignificant in these individual models.

| Variable | Time window 2 | Time window 3 | Time window 4 | Time window 5 | Time window 6 | Time window 7 | Time window 8 |
| --- | --- | --- | --- | --- | --- | --- | --- |
| (Intercept) | -39.1 *** | -31.6 *** | -27.5 *** | -19.9 *** | -13.8 *** | -11.5 *** | -11.7 *** |
| Leaf habit (evergreen) | 19.9 *** | 16.2 *** | 13.8 *** | 9.9 *** | 5.4 *** | 3.4 *** | 3.9 *** |
| Tree age | 0.1 ns | 1.7 ns | 1.2 ns | 1.6 ns | 0.8 ns | 1.2 ns | 0.1 ns |
| Tree size | -0.1 ns | 0.9 ns | 0.5 ns | -1.4 ns | -1 ns | -0.6 ns | -0.6 ns |
| Drought season (Spring and Summer) | 2.1 * | -0.3 ns | -3.1 ** | -2 * | -4.3 *** | -4.3 *** | -4.2 *** |
| Drought season (Summer) | 6 *** | 5.1 *** | 2.6 ** | 1.5 ns | -1.3 ns | -1.3 ns | -2.2 * |
| SPEI | 5.7 *** | 1 ns | -1 ns | 1.8 ns | -2.8 ** | -3.8 *** | -3.2 ** |
| MAT | -7.8 *** | -7.5 *** | -6.3 *** | -3.9 *** | -2.8 ** | -4 *** | -5 *** |
| MAP | 6.5 *** | 4.8 *** | 4.6 *** | 4 *** | 4.2 *** | 5.9 *** | 6.2 *** |
| Number of event years | 4.2 *** | 1.1 ns | 0.7 ns | 0.1 ns | 0.9 ns | -0.4 ns | -0.6 ns |
| Elevation | 6.2 *** | 6.8 *** | 6.2 *** | 6.4 *** | 4.9 *** | 4 *** | 3.7 *** |
| Year | 1.1 ns | 1.3 ns | 0.9 ns | -2.4 * | -0.5 ns | -0.7 ns | -0.6 ns |
| Leaf habit (evergreen):Tree age | -1.7 ns | -2.9 ** | -2.3 * | -2.1 * | -1.1 ns | -1.4 ns | -0.3 ns |
| Tree age:Number of event years | -1.5 ns | -2.6 ** | -0.9 ns | -1.4 ns | 0.2 ns | 0.1 ns | 3.2 ** |
| Leaf habit (evergreen):Drought season (Spring and Summer) | -2.9 ** | -1 ns | 2.7 ** | 2 * | 4.7 *** | 5.6 *** | 5.5 *** |
| Leaf habit (evergreen):Drought season (Summer) | -7.2 *** | -6.1 *** | -3.3 *** | -2.1 * | 1.4 ns | 2.9 ** | 3.8 *** |
| Leaf habit (evergreen):SPEI | -4.2 *** | -0.7 ns | 0.8 ns | -1.1 ns | 3.5 *** | 5.3 *** | 4.7 *** |
| Leaf habit (evergreen):MAT | 3.9 *** | 4.1 *** | 3.3 *** | 1.7 ns | 0.9 ns | 2.3 * | 3.2 ** |
| Leaf habit (evergreen):MAP | -3.1 ** | -1.9 ns | -2.4 * | -2.1 * | -2.5 * | -4.5 *** | -4.8 *** |
| Leaf habit (evergreen):Number of event years | -4.6 *** | -2.1 * | -2.1 * | -1.6 ns | -2.2 * | 0 ns | -0.1 ns |
| Number of event years:Elevation | -2.3 * | 0 ns | -3.7 *** | -1.2 ns | 0.4 ns | -1.1 ns | -0.4 ns |
| Leaf habit (evergreen):Year | -2.5 * | -2.1 * | -1.6 ns | 1.5 ns | -0.3 ns | -0.8 ns | -0.7 ns |
| Leaf habit (evergreen):Tree age:Number of event years | 4.2 *** | 5.4 *** | 2.7 ** | 2.6 * | 0.6 ns | 0.7 ns | -2.3 * |

**Table S4. Results from growth recovery (Rc) models considering individual time windows, ranging from 2 to 8 years**. T-values and significance levels are displayed (*** = *P* < 0.001; ** = *P* < 0.01; * = *P* < 0.05, ‘ns’ = nonsignificant effect). Positive t-values with significant effects are highlighted in green, negative t-values with significant effects are highlighted in red, and nonsignificant effects are highlighted in gray. For comparison purposes, the exact same list of variables and interactions as in the main models were retained, even if nonsignificant in these individual models.

| Variable | Time window 2 | Time window 3 | Time window 4 | Time window 5 | Time window 6 | Time window 7 | Time window 8 |
| --- | --- | --- | --- | --- | --- | --- | --- |
| (Intercept) | 17.6 *** | 20 *** | 19.8 *** | 13.4 *** | 9.5 *** | 8.5 *** | 9.3 *** |
| Leaf habit (evergreen) | -10.1 *** | -12.4 *** | -12.1 *** | -7.9 *** | -4.4 *** | -3 ** | -3.2 ** |
| Tree age | -3.5 *** | -8.5 *** | -6.7 *** | -5.2 *** | -3.7 *** | -3.7 *** | -2.9 ** |
| Tree size | -2.6 ** | -2 * | -1.8 ns | -1.3 ns | -2.4 * | -1.1 ns | -0.9 ns |
| Drought season (Spring and Summer) | 0.4 ns | -0.1 ns | 3 ** | 2.2 * | 4.1 *** | 3.4 *** | 4.5 *** |
| Drought season (Summer) | -3 ** | -8.1 *** | -6.1 *** | -1 ns | 0.6 ns | -0.3 ns | 0.4 ns |
| SPEI | -1.7 ns | -2.7 ** | -0.4 ns | -0.5 ns | 1.1 ns | 0.3 ns | 0.7 ns |
| MAT | 4.2 *** | 4.6 *** | 5 *** | 3.8 *** | 2.8 ** | 4.8 *** | 5.1 *** |
| MAP | -3.1 ** | -1.5 ns | -2.5 * | -3 ** | -3.3 ** | -5.5 *** | -4.9 *** |
| Number of event years | -5 *** | -1.8 ns | -1.6 ns | -0.8 ns | -2.4 * | -0.9 ns | -0.2 ns |
| Elevation | 2.9 ** | 2 * | 1 ns | -0.9 ns | -0.9 ns | -2.8 ** | -1.4 ns |
| Year | -2.8 ** | -7.1 *** | -7.5 *** | -2.3 * | -2.7 ** | -4.4 *** | -4.1 *** |
| Leaf habit (evergreen):Tree age | 3.6 *** | 8 *** | 6.1 *** | 4.4 *** | 3 ** | 2.9 ** | 2.2 * |
| Tree age:Number of event years | 4.9 *** | 8.5 *** | 6.3 *** | 4.6 *** | 3.5 *** | 3.7 *** | -0.1 ns |
| Leaf habit (evergreen):Drought season (Spring and Summer) | 0.9 ns | 1.5 ns | -2.6 ** | -1.7 ns | -4 *** | -4.3 *** | -5.8 *** |
| Leaf habit (evergreen):Drought season (Summer) | 4.3 *** | 8.9 *** | 6.7 *** | 2.7 ** | 0.3 ns | -0.1 ns | -1.6 ns |
| Leaf habit (evergreen):SPEI | 2.7 ** | 1.9 ns | -0.5 ns | -0.8 ns | -1.9 ns | -1.4 ns | -2.8 ** |
| Leaf habit (evergreen):MAT | -2.7 ** | -3.4 *** | -3.8 *** | -3 ** | -2 * | -3.7 *** | -4 *** |
| Leaf habit (evergreen):MAP | 1 ns | -0.3 ns | 1.2 ns | 2.1 * | 2.3 * | 4.3 *** | 3.9 *** |
| Leaf habit (evergreen):Number of event years | 4.8 *** | 1.1 ns | 0.6 ns | 0.1 ns | 1.7 ns | 0.2 ns | 0.1 ns |
| Leaf habit (evergreen):Elevation | -2.5 * | -1.9 ns | -1 ns | 0.6 ns | 0.5 ns | 2.4 * | 1.1 ns |
| Leaf habit (evergreen):Year | 2.1 * | 6.1 *** | 7.1 *** | 2.9 ** | 3.1 ** | 4.6 *** | 4 *** |
| Leaf habit (evergreen):Tree age:Number of event years | -5.2 *** | -7.9 *** | -5.1 *** | -3 ** | -2.4 * | -2.9 ** | 0.4 ns |

**Table S5. Results from growth resilience (Rs) models considering individual time windows, ranging from 2 to 8 years**. T-values and significance levels are displayed (*** = *P* < 0.001; ** = *P* < 0.01; * = *P* < 0.05, ‘ns’ = nonsignificant effect). Positive t-values with significant effects are highlighted in green, negative t-values with significant effects are highlighted in red, and nonsignificant effects are highlighted in gray. For comparison purposes, the exact same list of variables and interactions as in the main models were retained, even if nonsignificant in these individual models.

| Variable | Time window 2 | Time window 3 | Time window 4 | Time window 5 | Time window 6 | Time window 7 | Time window 8 |
| --- | --- | --- | --- | --- | --- | --- | --- |
| (Intercept) | -20 *** | -11.2 *** | -5.8 *** | -5.6 *** | -5.6 *** | -4.6 *** | -2.1 * |
| Leaf habit | 8.4 *** | 3.1 ** | -0.7 ns | 0.1 ns | 1.9 ns | 2.0 * | 0.8 ns |
| Tree age | -4.3 *** | -9.5 *** | -8.3 *** | -6.3 *** | -5.2 *** | -4.7 *** | -4.8 *** |
| Tree size | -2.8 ** | -1.1 ns | -1.4 ns | -2.8 ** | -3.6 *** | -1.9 ns | -1.6 ns |
| Drought season (Spring and Summer) | 2.4 * | 0.6 ns | 0.3 ns | -1.6 ns | 1.2 ns | 2.6 * | 2.1 * |
| Drought season (Summer) | 2.1 * | -4.4 *** | -6.4 *** | -1.2 ns | -0.1 ns | 0.1 ns | -1.8 ns |
| SPEI | 9.8 *** | -2.6 ** | -4.6 *** | -1.3 ns | -1.0 ns | 0.2 ns | -2.9 ** |
| MAT | -7.2 *** | -7.1 *** | -5.4 *** | -5.3 *** | -4.4 *** | -1.2 ns | -2.0 ns |
| MAP | 2.8 ** | 3.6 ** | 2.9 ** | 2.1 * | 1.3 ns | 0.7 ns | 1.9 ns |
| Number of event years | -1.8 ns | -1.3 ns | -1.7 ns | -1 ns | -1.9 ns | -2.0 * | -1.2 ns |
| Elevation | 6.2 *** | 5.3 *** | 3 ** | 0.9 ns | -0.7 ns | -0.9 ns | -0.4 ns |
| Year | -2.1 ns | -7.8 *** | -9.4 *** | -5.6 *** | -3.8 *** | -6.1 *** | -6.0 *** |
| Leaf habit:Tree age | 2.9 ** | 7.5 *** | 6.0 *** | 4.5 *** | 3.8 ** | 3.3 ** | 3.6 *** |
| Tree age:Number of event years | 3.9 *** | 7.7 *** | 7.1 *** | 4.8 *** | 6.1 *** | 5.1 *** | 3.9 *** |
| Leaf habit:Drought season (Spring and Summer) | -1.5 ns | -0.4 ns | -0.3 ns | 2.7 ** | -0.7 ns | -2.2 * | -2.4 * |
| Leaf habit:Drought season (Summer) | -1.7 ns | 4.1 *** | 6.5 *** | 2.8 ** | 1.6 ns | 1.6 ns | 2.2 * |
| Leaf habit:MAP | -1.9 ns | -2.7 * | -2.1 * | -1.0 ns | -0.7 ns | -0.5 ns | -1.6 ns |
| Leaf habit:Number of event years | 1.2 ns | -0.6 ns | -1.2 ns | -1.6 ns | -0.5 ns | 0.6 ns | 0.2 ns |
| Leaf habit:Elevation | -4 *** | -3.3 ** | -1.3 ns | 0.2 ns | 1.4 ns | 1.2 ns | -0.2 ns |
| Number of event years:Elevation | -1.5 ns | 0.5 ns | -2.3 * | 0.2 ns | -0.6 ns | -0.7 ns | -1.8 ns |
| Leaf habit:Year | -0.3 ns | 5.6 *** | 7.9 *** | 5.1 *** | 3.3 *** | 4.5 *** | 4.3 *** |
| Leaf habit:Tree age:Number of event years | -1.7 ns | -3.9 *** | -3.4 *** | -1.1 ns | -3.5 *** | -3.0 ** | -2.3 * |

**Table S6. Results from relative resilience (rel. Rs) models considering individual time windows, ranging from 2 to 8 years**. T-values and significance levels are displayed (*** = *P* < 0.001; ** = *P* < 0.01; * = *P* < 0.05, ‘ns’ = nonsignificant effect). Positive t-values with significant effects are highlighted in green, negative t-values with significant effects are highlighted in red, and nonsignificant effects are highlighted in gray. For comparison purposes, the exact same list of variables and interactions as in the main models were retained, even if nonsignificant in these individual models.

| Variable | Time window 2 | Time window 3 | Time window 4 | Time window 5 | Time window 6 | Time window 7 | Time window 8 |
| --- | --- | --- | --- | --- | --- | --- | --- |
| (Intercept) | 1558 *** | 1201.3 *** | 1115.7 *** | 815.2 *** | 557.8 *** | 441.5 *** | 390.3 *** |
| Leaf habit (evergreen) | -7.2 *** | -7.6 *** | -10.4 *** | -5.3 *** | -2.4 * | -2.3 * | -1.5 ns |
| Tree age | -4.8 *** | -9.1 *** | -7.8 *** | -5.9 *** | -5 *** | -4.9 *** | -5.2 *** |
| Tree size | -2.7 ** | -1.7 ns | -1.8 ns | -0.8 ns | -2.1 * | -0.8 ns | -0.5 ns |
| Drought season (Spring and Summer) | 2.3 * | 2.6 ** | 2.2 * | 1.6 ns | 1.9 ns | 1 ns | 1.5 ns |
| Drought season (Summer) | -1.4 ns | -5.6 *** | -7.7 *** | -0.5 ns | -0.2 ns | -1.8 ns | -2.2 * |
| SPEI | 0.3 ns | -3.1 ** | -2.8 ** | 0.1 ns | -0.5 ns | -2.1 * | -1.9 ns |
| MAT | 3.5 *** | 4.7 *** | 4.3 *** | 3 ** | 1.7 ns | 2.3 * | 2.6 ** |
| MAP | -8.8 *** | -6.5 *** | -3.5 *** | -0.7 ns | -1.8 ns | -2.5 * | -1.2 ns |
| Number of event years | -3 ** | -0.4 ns | -0.5 ns | -0.2 ns | -1 ns | -0.5 ns | 0.1 ns |
| Elevation | 3.2 ** | 2.3 * | 0.5 ns | -1.5 ns | -0.9 ns | -1.3 ns | -0.4 ns |
| Year | -2.6 * | -6.4 *** | -9.9 *** | -4.7 *** | -3.4 *** | -5.6 *** | -5.1 *** |
| Leaf habit (evergreen):Tree age | 4 *** | 7.7 *** | 6.1 *** | 4.3 *** | 3.8 *** | 3.8 *** | 4.3 *** |
| Tree age:Number of event years | 4.6 *** | 8 *** | 6.5 *** | 4.7 *** | 4.7 *** | 4.3 *** | 3 ** |
| Leaf habit (evergreen):Drought season (Spring and Summer) | -1.5 ns | -1.6 ns | -1.8 ns | -0.9 ns | -1.9 ns | -1.5 ns | -2.6 ** |
| Leaf habit (evergreen):Drought season (Summer) | 2.2 * | 6.1 *** | 8.2 *** | 2.3 * | 1.1 ns | 1.9 ns | 1.1 ns |
| Leaf habit (evergreen):SPEI | 1.1 ns | 2.6 ** | 1.8 ns | -1 ns | -0.2 ns | 1.7 ns | 0.1 ns |
| Leaf habit (evergreen):MAT | -1.8 ns | -3.5 *** | -3 ** | -2 * | -0.8 ns | -0.9 ns | -1.4 ns |
| Leaf habit (evergreen):Number of event years | 2.9 ** | -0.6 ns | -1.3 ns | -1.5 ns | -0.8 ns | -0.6 ns | -1 ns |
| Leaf habit (evergreen):Elevation | -2.6 * | -2 * | -0.3 ns | 1.3 ns | 0.5 ns | 0.9 ns | 0 ns |
| Leaf habit (evergreen):Year | 1.5 ns | 5.3 *** | 9.6 *** | 5.3 *** | 3.7 *** | 5.2 *** | 4.2 *** |
| Leaf habit (evergreen):Tree age:Number of event years | -3.6 *** | -5.8 *** | -4.1 *** | -1.9 ns | -2.8 ** | -2.8 ** | -1.9 ns |

**Table S7. Results from the species-specific linear mixed models (based on Eq. 2 in the main text) for each of the four resilience indices.** For fixed effects, t-values and significance levels are reported (ns: not significant; * P < 0.05; ** P < 0.01; *** P < 0.001). For random effects, significance was assessed using likelihood ratio tests comparing the full model to a reduced model without the random term of interest. At the bottom of the table, marginal R-squared values (reflecting variance explained by fixed effects only) and conditional R-squared values (reflecting variance explained by both fixed and random effects) are reported for each resilience index.

| **Effect** | **Type** | **Resistance (Rt)** | | **Recovery (Rc)** | | **Resilience (Rs)** | | **Relative resilience (Rel. Rs)** | |
| --- | --- | --- | --- | --- | --- | --- | --- | --- | --- |
|  |  | **T-value** | **signif.** | **T-value** | **signif.** | **T-value** | **signif.** | **T-value** | **signif.** |
| (Intercept) | - | -9.5 | *** | 9.0 | *** | 0.7 | ns | 810.1 | *** |
| speciesABIEBAL | Fixed | -2.3 | * | 1.0 | ns | -1.4 | ns | -0.4 | ns |
| speciesABIELAS | Fixed | 1.5 | ns | -0.6 | ns | 0.8 | ns | -0.6 | ns |
| speciesACERRUB | Fixed | -6.9 | *** | 6.6 | *** | 0.3 | ns | 4.2 | *** |
| speciesBETUPAP | Fixed | -6.4 | *** | 2.0 | * | -4.3 | *** | 0 | ns |
| speciesLARILAR | Fixed | -9.1 | *** | -0.3 | ns | -10 | *** | -1.8 | ns |
| speciesLARILYA | Fixed | -7.5 | *** | 7.1 | *** | 0.3 | ns | 3.1 | ** |
| speciesPICEENG | Fixed | 0.7 | ns | -0.4 | ns | 0.1 | ns | -1.1 | ns |
| speciesPICEGLA | Fixed | -3 | ** | 0.6 | ns | -2.7 | ** | -1.2 | ns |
| speciesPICEMAR | Fixed | 0.1 | ns | -2.8 | ** | -3.3 | *** | -4.1 | *** |
| speciesPICESIT | Fixed | 0.9 | ns | -2.6 | * | -1.5 | ns | -3.1 | ** |
| speciesPINUALB | Fixed | -3.8 | *** | 3.9 | *** | 0.4 | ns | 0.3 | ns |
| speciesPINUBAN | Fixed | -1.9 | ns | -4.0 | *** | -6.7 | *** | -5.9 | *** |
| speciesPINUCON | Fixed | -2 | * | -2.4 | * | -5 | *** | -4.3 | *** |
| speciesPINURES | Fixed | -1.3 | ns | -2.4 | * | -4.3 | *** | -3.5 | *** |
| speciesPINUSTR | Fixed | -3.4 | *** | 1.1 | ns | -2.4 | * | -0.2 | ns |
| speciesPOPUBAL | Fixed | -7.2 | *** | 5.8 | *** | -0.8 | ns | 1.9 | ns |
| speciesPOPUTRE | Fixed | -14.2 | *** | 8.5 | *** | -5 | *** | 3 | ** |
| speciesPSEUMEN | Fixed | -3.5 | *** | 0.3 | ns | -3.4 | *** | -1.8 | ns |
| speciesTHUJOCC | Fixed | 1.5 | ns | -0.3 | ns | 1.1 | ns | -1.7 | ns |
| speciesTHUJPLI | Fixed | -3.1 | ** | 0.9 | ns | -2.2 | * | -1.5 | ns |
| speciesTSUGHET | Fixed | -4.2 | *** | 1.1 | ns | -3.1 | ** | -1 | ns |
| speciesTSUGMER | Fixed | 2.5 | * | -3.4 | *** | -1.3 | ns | -2.1 | * |
| Ring Age | Fixed | -19.2 | *** | -20.3 | *** | -43.4 | *** | -35.9 | *** |
| Tree size | Fixed | -0.4 | ns | -5.6 | *** | -6.9 | *** | -5.3 | *** |
| Time window | Random | - | *** | - | *** | - | *** | - | *** |
| TimingGrowDecl | Random | - | *** | - | *** | - | *** | - | *** |
| Plot ID | Random | - | *** | - | *** | - | *** | - | *** |
|  |  |  |  |  |  |  |  |  |  |
| Conditional R^2^ |  | 0.36 | | 0.39 | | 0.31 | | 0.47 | |
| Marginal R^2^ |  | 0.09 | | 0.07 | | 0.02 | | 0.04 | |

**Methods S1: Assessing the impact of autocorrelation on tree selection and drought detection.**

Autocorrelation inherently smooths out fluctuations in tree-ring data by “carrying over” the effect of previous years’ plant productivity on current growth, potentially reducing the impact of drought in a given growth year and spreading that impact across multiple years (Esper et al. 2013). This can result in a reduction in the BAI during the current growth year of interest appearing less severe, potentially leading to overlooked or underestimated drought impacts. To verify whether the presence of autocorrelation in BAI time series affected our capacity to detect drought-induced growth reductions and, consequently, the inclusion of specific trees in our analyses, we performed Cohen’s *d* tests on the first-order autocorrelation (AR1) estimated for each tree using the *acf* function of the *stats* R-package. Cohen’s *d* test measures the effect size magnitude by calculating the difference between the means of two groups divided by the standard deviation, providing a size-independent metric (Sullivan & Feinn, 2012). The two groups consisted of trees with at least one detected drought-stress event and those without any detected event that were subsequently excluded. We used the *cohens_d* function from the *rstatix* package (Kassambara, 2023). Analyses were conducted independently for each time window, resulting in |d| < 0.1 ("negligible"). This finding indicates that autocorrelation did not influence the exclusion of trees from our analyses (Figure S4, Table S1). Therefore, the individuals included in our study could be considered representative of the available samples, ensuring that there is no bias associated with defining drought-stress event years on the basis of a negative SPEI and reductions in growth.

**Methods S2: Assessing resilience indices in relation to long-term tree growth**

Linear mixed models were used to evaluate the relationships between the four resilience indices and the cumulative growth performance of trees over their lifespan, as represented by their diameter at breast height (DBH). These models were fitted separately for each species, with the log-transformed, tree-level average values of resilience indices as response variables, and diameter at breast height - a proxy for the growth performance of individual trees - as the explanatory variable. The time window length and plot identity were included as random effects. In total, 92 models were fitted (23 species × 4 resilience indices). Models’ structure was as follows:

| Index_ij_ ~ DBH_ij_ + (Plot ID, time window) + ε | Eq. S1 |
| --- | --- |

where Index_ij_ refers to the mean, log-transformed, values of each of the four resilience indices for a tree i in a plot j, DBH_ij_ is the diameter at breast height of a tree i in a plot j, as measured at the time of sampling, (Plot ID, time windows) is the random effect of the plot identity and time window length, and ε refers to the residual error term. We used t-statistics to evaluate the strength and direction of these relationships.

**Supplementary References**

Bose, A.K., Doležal, J., Scherrer, D., Altman, J., Ziche, D., Martínez-Sancho, E., Bigler, C., Bolte, A., Colangelo, M., Dorado-Liñán, I., 2024. Revealing legacy effects of extreme droughts on tree growth of oaks across the Northern Hemisphere. Sci. Total Environ. 926, 172049.

Bose, A.K., Scherrer, D., Camarero, J.J., Ziche, D., Babst, F., Bigler, C., Bolte, A., Dorado-Liñán, I., Etzold, S., Fonti, P., Forrester, D.I., Gavinet, J., Gazol, A., de Andrés, E.G., Karger, D.N., Lebourgeois, F., Lévesque, M., Martínez-Sancho, E., Menzel, A., Neuwirth, B., Nicolas, M., Sanders, T.G.M., Scharnweber, T., Schröder, J., Zweifel, R., Gessler, A., Rigling, A., 2021. Climate sensitivity and drought seasonality determine post-drought growth recovery of Quercus petraea and Quercus robur in Europe. Sci. Total Environ. 784, 147222. <https://doi.org/10.1016/j.scitotenv.2021.147222>

Bottero, A., Forrester, D.I., Cailleret, M., Kohnle, U., Gessler, A., Michel, D., Bose, A.K., Bauhus, J., Bugmann, H., Cuntz, M., Gillerot, L., Hanewinkel, M., Lévesque, M., Ryder, J., Sainte-Marie, J., Schwarz, J., Yousefpour, R., Zamora-Pereira, J.C., Rigling, A., 2021. Growth resistance and resilience of mixed silver fir and Norway spruce forests in central Europe: Contrasting responses to mild and severe droughts. Glob. Change Biol. 27, 4403–4419. <https://doi.org/10.1111/gcb.15737>

Fang O, Zhang Q-B. Tree resilience to drought increases in the Tibetan Plateau. Glob Change Biol. 2019; 25: 245–253. <https://doi.org/10.1111/gcb.14470>

Gessler, A., Bottero, A., Marshall, J., Arend, M., 2020. The way back: recovery of trees from drought and its implication for acclimation. New Phytol. 228, 1704–1709. <https://doi.org/10.1111/nph.16703>

Groover, A., Holbrook, N.M., Polle, A., Sala, A., Medlyn, B., Brodersen, C., Pittermann, J., Gersony, J., Sokołowska, K., Bogar, L., McDowell, N., Spicer, R., David-Schwartz, R., Keller, S., Tschaplinski, T.J. and Preisler, Y. (2025), Tree drought physiology: critical research questions and strategies for mitigating climate change effects on forests. New Phytol. <https://doi.org/10.1111/nph.20326>

Hasselquist, N.J., Allen, M.F. & Santiago, L.S. Water relations of evergreen and drought-deciduous trees along a seasonally dry tropical forest chronosequence. Oecologia 164, 881–890 (2010). <https://doi.org/10.1007/s00442-010-1725-y>

Isaac-Renton, M., Montwé, D., Hamann, A., Spiecker, H., Cherubini, P., Treydte, K., 2018. Northern forest tree populations are physiologically maladapted to drought. Nat. Commun. 9, 1–9. <https://doi.org/10.1038/s41467-018-07701-0>

Jiang, Y., Marchand, W., Rydval, M., Matula, R., Janda, P., Begović, K., Thom, D., Fruleux, A., Buechling, A., Pavlin, J., Nogueira, J., Dušátko, M., Málek, J., Kníř, T., Veber, A., Svoboda, M., 2024. Drought resistance of major tree species in the Czech Republic. Agric. For. Meteorol. 348, 109933. <https://doi.org/10.1016/j.agrformet.2024.109933>

Kaproth, M.A., Fredericksen, B.W., González‐Rodríguez, A., Hipp, A.L., Cavender‐Bares, J., 2023. Drought response strategies are coupled with leaf habit in 35 evergreen and deciduous oak ( Quercus ) species across a climatic gradient in the Americas. New Phytol. 239, 888–904. <https://doi.org/10.1111/nph.19019>

Kassambara, A., 2023. rstatix: Pipe-Friendly Framework for Basic Statistical Tests.

Li, Y., Zhang, W., Schwalm, C.R. et al. Widespread spring phenology effects on drought recovery of Northern Hemisphere ecosystems. Nat. Clim. Chang. 13, 182–188 (2023). <https://doi.org/10.1038/s41558-022-01584-2>

Li, X., Piao, S., Wang, K., Wang, X., Wang, T., Ciais, P., Chen, A., Lian, X., Peng, S., Peñuelas, J., 2020. Temporal trade-off between gymnosperm resistance and resilience increases forest sensitivity to extreme drought. Nat. Ecol. Evol. 1–9. <https://doi.org/10.1038/s41559-020-1217-3>

Lucas-Borja, M.E., Bose, A.K., Andivia, E., Candel-Pérez, D., Plaza-Álvarez, P.A., Linares, J.C., 2021. Assessing Tree Drought Resistance and Climate-Growth Relationships under Different Tree Age Classes in a Pinus nigra Arn. ssp. salzmannii Forest. Forests 12, 1161. <https://doi.org/10.3390/f12091161>

Rodriguez-Zaccaro FD, Groover A. Wood and water: How trees modify wood development to cope with drought. Plants, People, Planet, 2019; 1: 346–355. <https://doi.org/10.1002/ppp3.29>

Ruehr NK, Grote R, Mayr S, Arneth A, Beyond the extreme: recovery of carbon and water relations in woody plants following heat and drought stress, Tree Physiology, Volume 39, Issue 8, August 2019, Pages 1285–1299, <https://doi.org/10.1093/treephys/tpz032>

Schwarz, J., Skiadaresis, G., Kohler, M., Kunz, J., Schnabel, F., Vitali, V., Bauhus, J., 2020. Quantifying Growth Responses of Trees to Drought—a Critique of Commonly Used Resilience Indices and Recommendations for Future Studies. Curr. For. Rep. 6, 185–200. <https://doi.org/10.1007/s40725-020-00119-2>

Serra-Maluquer, X., Gazol, A., Anderegg, W.R.L., Martínez-Vilalta, J., Mencuccini, M., Camarero, J.J., 2022. Wood density and hydraulic traits influence species’ growth response to drought across biomes. Glob. Chang. Biol.

Song, Y., Sterck, F., Zhou, X., Liu, Q., Kruijt, B. and Poorter, L. (2022), Drought resilience of conifer species is driven by leaf lifespan but not by hydraulic traits. New Phytol, 235: 978-992. <https://doi.org/10.1111/nph.18177>

Stone GN, Nee S, Felsenstein J. Controlling for non-independence in comparative analysis of patterns across populations within species. Philos Trans R Soc Lond B Biol Sci. 2011 May 12;366(1569):1410-24. doi: 10.1098/rstb.2010.0311. PMID: 21444315; PMCID: PMC3081573.

Stralberg, D., Arseneault, D., Baltzer, J.L., Barber, Q.E., Bayne, E.M., Boulanger, Y., Brown, C.D., Cooke, H.A., Devito, K., Edwards, J., Estevo, C.A., Flynn, N., Frelich, L.E., Hogg, E.H., Johnston, M., Logan, T., Matsuoka, S.M., Moore, P., Morelli, T.L., Morissette, J.L., Nelson, E.A., Nenzén, H., Nielsen, S.E., Parisien, M.-A., Pedlar, J.H., Price, D.T., Schmiegelow, F.K., Slattery, S.M., Sonnentag, O., Thompson, D.K., Whitman, E., 2020. Climate-change refugia in boreal North America: what, where, and for how long? Front. Ecol. Environ. 18, 261–270. <https://doi.org/10.1002/fee.2188>

Sullivan, G.M., Feinn, R., 2012. Using Effect Size—or Why the P Value Is Not Enough. J. Grad. Med. Educ. 4, 279–282. <https://doi.org/10.4300/JGME-D-12-00156.1>

Trugman, A.T., Detto, M., Bartlett, M.K., Medvigy, D., Anderegg, W.R.L., Schwalm, C., Schaffer, B. and Pacala, S.W. (2018), Tree carbon allocation explains forest drought-kill and recovery patterns. Ecol Lett, 21: 1552-1560. <https://doi.org/10.1111/ele.13136>

Zheng, T., Martínez-Vilalta, J., García-Valdés, R., Gazol, A., Camarero, J.J., Mu, C., Mencuccini, M., 2023. Growth plasticity of conifers did not avoid declining resilience to soil and atmospheric droughts during the 20th century. For. Ecosyst. 10, 100107. <https://doi.org/10.1016/j.fecs.2023.100107>

Zheng, T., Martínez-Vilalta, J., García-Valdés, R., Gazol, A., Camarero, J.J., Mencuccini, M., 2021. Disentangling biology from mathematical necessity in twentieth-century gymnosperm resilience trends - Nature Ecology & Evolution. Nat. Ecol. Evol. 5, 733–735. <https://doi.org/10.1038/s41559-021-01436-w>
